# The Polyunsaturated Fatty Acids Arachidonic Acid and Docosahexaenoic Acid Induce Mouse Dendritic Cells Maturation but Reduce T-Cell Responses *In Vitro*

**DOI:** 10.1371/journal.pone.0143741

**Published:** 2015-11-30

**Authors:** Johan A. Carlsson, Agnes E. Wold, Ann-Sofie Sandberg, Sofia M. Östman

**Affiliations:** 1 Department of Infectious Diseases, Institute of Biomedicine, the Sahlgrenska Academy, University of Gothenburg, Gothenburg, Sweden; 2 Divisions of Food and Nutrition Science, Department of Biology and Biological Engineering, Chalmers University of Technology, Gothenburg, Sweden; Universidade Federal do Rio de Janeiro, BRAZIL

## Abstract

Long-chain polyunsaturated fatty acids (PUFAs) might regulate T-cell activation and lineage commitment. Here, we measured the effects of omega-3 (n-3), n-6 and n-9 fatty acids on the interaction between dendritic cells (DCs) and naïve T cells. Spleen DCs from BALB/c mice were cultured *in vitro* with ovalbumin (OVA) with 50 μM fatty acids; α-linolenic acid, arachidonic acid (AA), eicosapentaenoic acid (EPA), docosahexaenoic acid (DHA), linoleic acid or oleic acid and thereafter OVA-specific DO11.10 T cells were added to the cultures. Fatty acids were taken up by the DCs, as shown by gas chromatography analysis. After culture with arachidonic acid or DHA CD11c^+^ CD11b^+^ and CD11c^+^ CD11b^neg^ DCs expressed more CD40, CD80, CD83, CD86 and PDL-1, while IA^d^ remained unchanged. However, fewer T cells co-cultured with these DCs proliferated (CellTrace Violet^low^) and expressed CD69 or CD25, while more were necrotic (7AAD^+^). We noted an increased proportion of T cells with a regulatory T cell (Treg) phenotype, i.e., when gating on CD4^+^ FoxP3^+^ CTLA-4^+^, CD4^+^ FoxP3^+^ Helios^+^ or CD4^+^ FoxP3^+^ PD-1^+^, in co-cultures with arachidonic acid- or DHA-primed DCs relative to control cultures. The proportion of putative Tregs was inversely correlated to T-cell proliferation, indicating a suppressive function of these cells. With arachidonic acid DCs produced higher levels of prostaglandin E_2_ while T cells produced lower amounts of IL-10 and IFNγ. In conclusion arachidonic acid and DHA induced up-regulation of activation markers on DCs. However arachidonic acid- and DHA-primed DCs reduced T-cell proliferation and increased the proportion of T cells expressing FoxP3, indicating that these fatty acids can promote induction of regulatory T cells.

## Introduction

Lymphoid organs are embedded in fat [1] and fatty acids, especially long-chain polyunsaturated fatty acids (PUFAs) have immunoregulatory functions via several mechanisms. They are incorporated into cell membranes and affect fluidity, formation of lipid rafts and protein configuration and are thereby modulating cell communication [2] but they also affect intracellular signaling. Fatty acids diffuse through the membrane freely, or via transporters, bind to cytoplasmic receptors termed fatty acid binding proteins and translocate to the nucleus, where they affect gene transcription. Lastly, some PUFAs are precursors of lipid mediators [3], which participate in inflammatory processes and also affect acquired immune cells. For example, prostaglandins are potent inhibitors of T-cell proliferation [4]. The most prominent effect of PUFAs is inhibited T-cell proliferation [5–12], particularly that of Th1 cells [13]. In general, the longer chains and the higher degree of unsaturation, the stronger inhibitory effect [10].

Antigen presenting cells, such as dendritic cells (DCs), initiate and regulate T-cell responses. DCs can have myeloid or lymphoid origin and these subsets differ in phenotype, localization, and function. In mice, simplified, myeloid DCs are CD11b^+^ CD8^-^ while lymphoid DCs are CD11b^-^ CD8^+^ DEC-205^+^ [14]. Both subsets express high levels of CD11c, MHC class II, CD86 and CD40 [15]. The heterogeneity of DCs makes it difficult to assign fixed functions to the subsets [16], but in general CD11b^+^ DCs present MHC class II-restricted antigens to CD4^+^ T cells [14], inducing a proliferative response [17]. On the contrary lymphoid CD8^+^ DCs induce a limited CD4^+^ T cell response, associated with apoptosis [18], as well as Th1 differentiation [19].

Presentation of antigen to naïve T cells results in activation or tolerance, depending on interaction of MHC molecule-TCR complex interaction, expression of costimulatory molecules, cell adhesion and cytokine milieu. Mature DCs express the glycoprotein CD83, related to the B7 ancestral family [20]. Costimulatory molecules on DCs include CD80 (B7-1) and CD86 (B7-2) that bind to CD28 on T cells, inducing T-cell activation and proliferation. However, CD80 and CD86 can also bind to CTLA-4 (CD152) [21], which inhibits T cell IL-2 secretion and proliferation [22]. Programmed cell death ligand 1 (PDL-1/CD274) on DCs inhibits T-cell activation and proliferation through interaction with programmed death-1 (PD-1, PDCD1/CD279) on T cells [23]. PD-1 is involved in regulation of peripheral tolerance and autoimmunity and the PD-1: PDL pathway promotes maturation of naïve T cells into FoxP3^+^ CD4^+^ regulatory T cells (Tregs) [24].

Long-chain PUFAs affect cytokine secretion and expression of costimulatory molecules on DCs [25]. In general fish oil and n-3 PUFAs reduce costimulatory molecules and antigen-presentation capacity, measured as subsequent T-cell activation [26–30]. The effects vary between different fatty acids, also between different n-3 PUFAs [31], dose and exposure time [5] and maturation stage of the DCs [32]. In this study, the immunoregulatory effects of fatty acids were tested by *in vitro* culture of murine CD11c^+^ DCs with free fatty acids. We evaluated DC phenotype, ability of fatty acid-primed DCs to activate T cells as well as subsequent T-cell phenotype.

## Material and Methods

### Animals

Male BALB/c mice (Charles River, Sulzfeld, Germany) were 6–8 weeks old when used to collect dendritic cells. Male DO11.10 H-2d [OVA T-cell receptor transgenic] BALB/c mice were the source of OVA-specific naïve T cells. They were bred at the animal facility at the University of Gothenburg under standard conditions. The study was carried out in accordance with recommendations from the Swedish board of agriculture and approved by the regional ethical committee (Göteborgs djurförsöksetiska nämnd, permit number: 365-2011/68-2012). Mice were sacrificed by cervical dislocation.

### Dendritic cell: T cell co-culture

The experimental design is shown in S1 Fig. Spleen DCs from mice were cultured in medium supplemented with fatty acids, see below, and the model antigen OVA for 3 days. On day 3 DCs were analyzed by flow cytometry for cell surface molecules (CD11b, CD11c, CD40, CD83, CD86, MHC class II IA^d^ and PDL-1). Alternatively CellTrace^™^-stained OVA-specific CD4^+^ T cells, isolated from DO11.10 mice, were added and co-cultured with the DCs for another 6 days. Thereafter T-cell proliferation and phenotype (expression of CD25, CD69, CTLA-4, FoxP3, Helios and PD-1) were determined by flow cytometry.

For DCs, eight mice were sacrificed and spleens removed and prepared into single cell suspension. CD11c^+^ cells were isolated with the MACS cell separation system (N418, Miltenyi Biotec GmbH, Bergisch Gladbach, Germany) and LS columns, according to the manufacturer’s instructions. CD11c^+^ cells (5x10^4^) were cultured in 96-well plates (Zellkultur Testplatte 96U, TPP) in Iscove’s modified Dulbecco’s medium (Sigma-Aldrich Co., St Louis, MO) supplemented with 10% fetal bovine serum, 1% β-mercaptoethanol (4 mM solution), 1% L-glutamine (200 mM solution, Sigma-Aldrich) and 0.1% gentamicin (50 mg/ml solution, GIBCO/Invitrogen, Eugene, OR). Fatty acids were dissolved in ethanol and added to DC cultures in a final concentration of 50 μM, a physiological concentration in plasma [33, 34], within a range of concentrations that have been used in previous *in vitro* experiments [5, 8, 12]. The final ethanol concentration in the cell cultures was max 1%, when ethanol alone was used as control. The following fatty acids were used; 18:1 n-9 oleic acid (OA), 18:2 n-6 linoleic acid (LA), 18:3 n-3 α-linolenic acid (ALA), 20:4 n-6 arachidonic acid (AA), 20:5 n-3 eicosapentaenoic acid (EPA) and 22:6 n-3 DHA were used (all from Sigma-Aldrich with purity ≥98%). Purity of stock solutions was tested by gas chromatography-mass spectrometry analysis, and found to be > 90% (S2 Fig). Stock solutions of fatty acids as well as cell culture medium were tested for presence of staphylococcal enterotoxin with the SET-RPLA kit (Oxoid Ltd, Basingstoke, UK) and found to be endotoxin free. OVA (grade III, Sigma-Aldrich) was added to a final concentration of 0.5 μg/μl in cell cultures later used for T-cell analysis. For blocking experiments purified anti-mouse CD83 (HB15, clone Michel-17) or PD-1 (CD279) (clone RMP1-14) antibodies were used in a final concentration of 10 μg/ml (both from eBioscience Inc., San Diego, CA). After 3 days the DCs were either harvested for FACS analysis of phenotype or further co-cultured with OVA-specific T cells (5x10^4^, giving a DC: T cell ratio of 1:1). Notably, DCs were washed and medium renewed before co-culture with T cells to avoid direct stimulation from fatty acids on T cells. Responder OVA-specific DO11.10 T cells were isolated and purified from spleens using the CD4^+^ T cell isolation kit II (Miltenyi Biotec GmbH) according to the manufacturer’s instructions. Isolated T cells were stained with CellTrace^™^ Violet (CellTrace^™^ Violet Cell Proliferation Kit, Molecular Probes/Invitrogen) in a final concentration of 5 μM, prior to addition to the CD11c^+^ cell cultures. After 6 days of co-culture T cells were harvested for flow cytometry analysis of proliferation and phenotype.

### Gas chromatography analysis of fatty acid content in dendritic cells

Cultured DCs were pooled to 4–8*10^6^ cells and washed four times with PBS. After complete evaporation of PBS, 4 μg of an internal fatty acid standard, 17:0 in phospholipid form (Larodan AB, Malmö, Sweden), dissolved in dichloromethane was added to the samples. Total lipids were extracted with 5 ml 2:1 chloroform-methanol solution for 1 h. 2 ml milliQ-H_2_O were added and phases separated by centrifugation. The non-polar phase was transferred to a new tube before liquid evaporation. Fatty acids were methylated for 1 h at 75°C using 1 ml 10% acetyl chloride in methanol and 1 ml toluene. 1 ml milliQ-H_2_O and 5 ml petroleum ether were added. The non-polar phase was transferred to a new tube before liquid evaporation. The fatty acid methyl esters (FAME) were dissolved in 150 μl iso-octane and separated on a Agilent Technologies 7890A GC with a 5975C MSD Triple-Axis detector (Agilent Technologies, Inc., Santa Clara, CA), using a VF-WAXms column (30 m x 0,25 mm, film 0,25 μm; Agilent Technologies). The fatty acids were identified with the MSD ChemStation software version E.02.01.1177 (Agilent Technologies) and peaks identified using the GLC-463 reference standard (Nu-Chek Prep, Inc., Elysian, MN).

### Flow cytometry

In brief, the procedure for flow cytometry was as follows. Cell cultures were centrifuged and resuspended in FACS wash buffer (prepared in-house) with a 1:100 solution of Fc-receptor block (anti-Mouse CD16/CD32 Purified, clone 93, eBioscience) and appropriate antibodies, diluted 1:100. Samples were incubated at 4°C for 20 min and washed twice prior to flow cytometry analysis. For intracellular staining of FoxP3, Helios and CTLA-4, the Foxp3/Transcription Factor Staining Buffer Set (eBioscience) was used. The last wash was followed by addition of permeabilization solution and samples were incubated at 4°C for 30 min. After centrifugation and 2x wash, cells were resuspended in permeabilization buffer. Fc-receptor block and antibodies (1:50) were added. After incubation, cells were washed twice in permeabilization buffer, followed by addition of FACS wash buffer and flow cytometry analysis.

Dendritic cells were analyzed for CD11b (conjugated with PerCP-Cy5.5, clone M1/70), CD11c (APC, clone HL3), CD80 (PE, clone 16-10A1), CD86 (Horizon V450, clone GL1), MHC class II IA^d^ (FITC, clone AMS-32.1) (all from BD Biosciences, San Jose, CA), CD40 (PE, clone 3/23), CD83 (FITC, clone Michel-19) and PDL-1 (Brilliant Violet 421, clone 10F.9G2) (all from BioLegend, San Diego, CA). T cells were analyzed for proliferation with CellTrace^™^ Violet or CellTrace^™^ CFSE stain, CD25 (PerCP-Cy5.5, clone PC61), CD69 (PerCP-Cy5.5, clone H1.2F3), CTLA-4 (PE, clone UC10-4F10-11) (all from BD Biosciences), DO11.10 TCR (FITC, clone KJ1-26), Helios (PerCP-Cy5.5, clone 22F6), PD-1 (APC, clone RMP1-30 (all from BioLegend) as well as CD4 (FITC, clone RM4-4), FoxP3 (APC, clone FJK-16s, eBioscience). Cell viability at the end of culture was analyzed with Annexin V (FITC, BioLegend) and 7-aminoactinomycin (7AAD) (BD Biosciences) in Annexin V binding buffer (BD Biosciences) according to the manufacturer’s instructions. Cells were acquired using FACS CantoII (BD Biosciences) and analyzed with FACSDiva (v. 6 or 8, BD Biosciences) and FlowJo (v. 7.6.5, TreeStar Inc., Ashland, OR) software. Gating was based on “fluorescence minus one” (FMO) samples; controls that include all of the antibody conjugates present in the test samples except one. For each marker gates were set likewise for all samples in order to enable comparison between samples.

### Cytokines, soluble CD83 and prostaglandin E_2_ in cell culture supernatant

Cytokines, IL-10, IL-12 p70, IFNγ and TGF-β1, were analyzed with DuoSet ELISA (R&D Systems, Abingdon, UK), soluble CD83 (sCD83) was analyzed with ELISA kit (Cloud-Clone Corp., Houston, TX) and prostaglandin E_2_ (PGE_2_) was analyzed with Parameter Assay Kit (R&D Systems) all according to instructions. A VERSAmax tunable microplate reader with the SoftMax^®^ Pro 5 software (Molecular Devices Corp., Sunnyvale, CA) were used for absorbance measurement.

### Statistical analysis

Data was tested for normality with the D'Agostino & Pearson omnibus normality test. Parametric one-way ANOVA or non-parametric Kruskal-Wallis test was used to test statistical mean difference compared to control, ethanol but no fatty acid, with correction for multiple comparisons. For correlation analysis the parametric Pearson correlation test or the non-parametric Spearman correlation test was used. In blocking experiments, the parametric t test or the non-parametric Mann Whiney U-test was used for comparison between blocking and no blocking. All tests were performed two-tailed. Statistical significance levels was set at p≤0.05 (*), p≤0.01 (**) p≤0.001 (***) and p≤0.0001 (****). For tests with all seven stimuli and eight mice only p-values below 0.01 were considered statistically significant.

## Results

### The supplemented fatty acids were taken up by the dendritic cells

**Fig 1:**
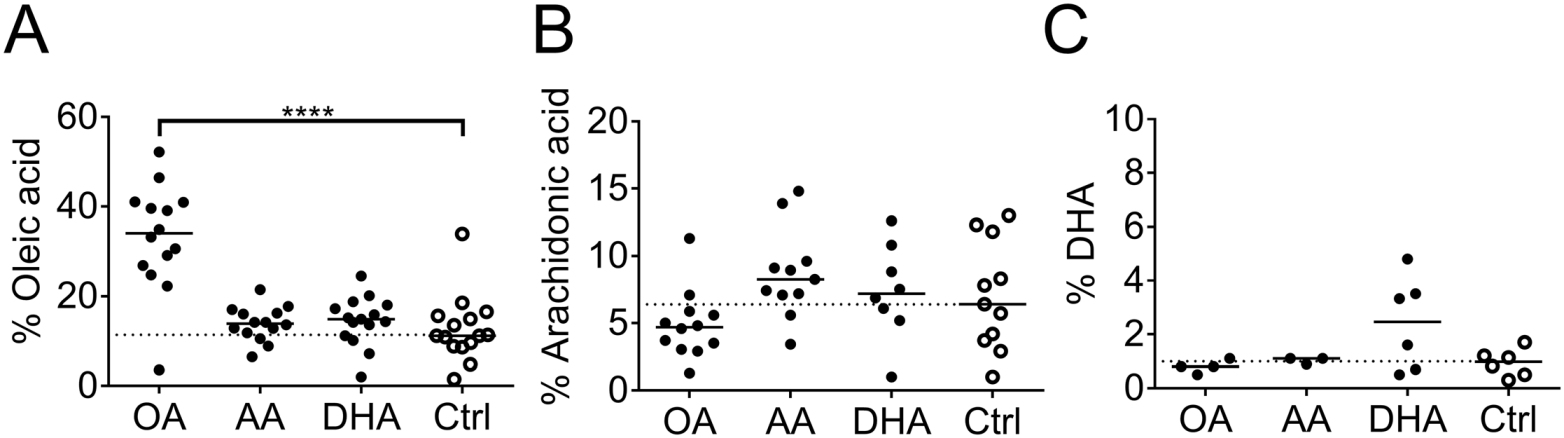
Fatty acid uptake by dendritic cells (DCs). DC cultures were supplemented with fatty acids (50 μM); arachidonic acid (AA), docosahexaenoic acid (DHA) or oleic acid (OA) for 3 days and thereafter the cells were analyzed by gas chromatography. The proportion of (A) oleic acid, (B) arachidonic acid and (C) DHA of all lipid content in the cells. Black dots denote samples supplemented with fatty acid while white dots with black borders denote control (ethanol only). Horizontal solid black lines show median value. The median from the control group has been extended with a dotted line for easy comparison to the other groups. Statistical mean difference was compared to the control group. p-values: ** <0.01, *** <0.001, **** <0.0001.

CD11c^+^ DCs, isolated from the spleen of BALB/c mice, were cultured for 3 days with different fatty acids in the cell culture medium. Supplementation of arachidonic acid, DHA or oleic acid in the culture medium was reflected in the fatty acid content of the DCs. When measured as proportion of the cells’ total amounts of fatty acids, the levels of oleic acid increased, from mean 12.7% to 33.2%, (p< 0.0001, Fig 1A) when added to the cell culture medium. Arachidonic acid changed from mean 7.0% to 8.7%, (p = 0.5253, Fig 1B) and DHA from mean 0.9% to 2.4%, (p = 0.7716, Fig 1C) but these changes did not reach statistical significance.

### No change in proportion of CD11c^+^CD11b^+/neg^ DCs after fatty acid supplementation

**Fig 2:**
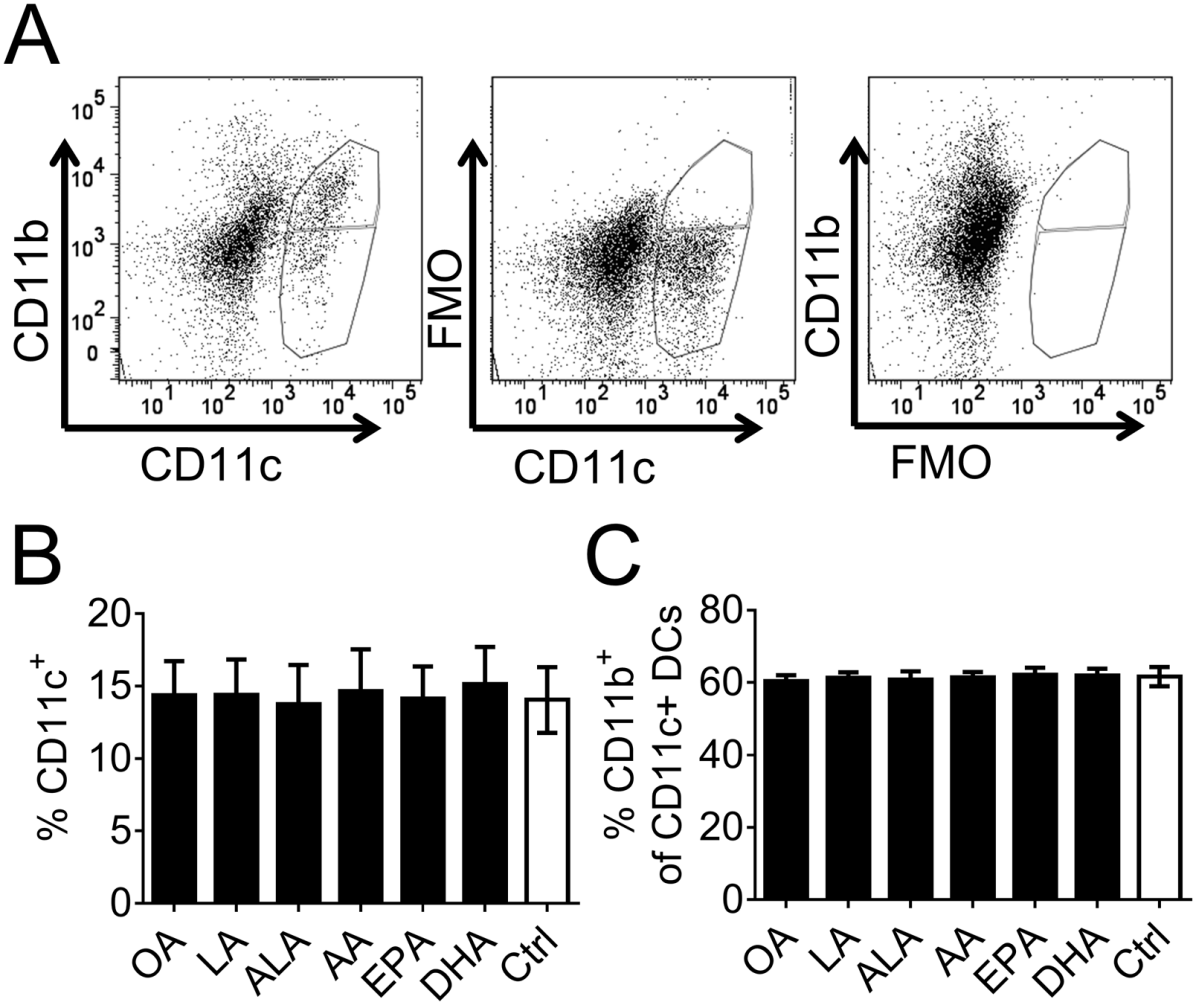
Distribution of dendritic cells (DCs) in fatty-acid supplemented cell cultures. DC cultures were supplemented with fatty acids (50 μM); α-linolenic acid (ALA), arachidonic acid (AA), eicosapentaenoic acid (EPA), docosahexaenoic acid (DHA), linoleic acid (LA) or oleic acid (OA) for 3 days and thereafter DCs were analyzed by flow cytometry. (A) Dot plots showing gating strategy for CD11c^+^ CD11b^neg^ (lower gate) and CD11c^+^ CD11b^+^ (upper gate) DCs based on FMO (fluorescence minus one) samples. To the right a representative sample, in the middle FMO for CD11b and to the right FMO for CD11c. (B) Proportion of CD11c^+^ DCs, both CD11b^neg^ and CD11b^+^, in fatty-acid supplemented cell cultures. (C) Proportion of CD11b^+^ cells within the CD11c^+^ population shown in Fig 2B. Black bars denote samples supplemented with fatty acid while white bar with black border denotes control (ethanol only). For each group n = 8. Error bars show standard deviation.

**Fig 3:**
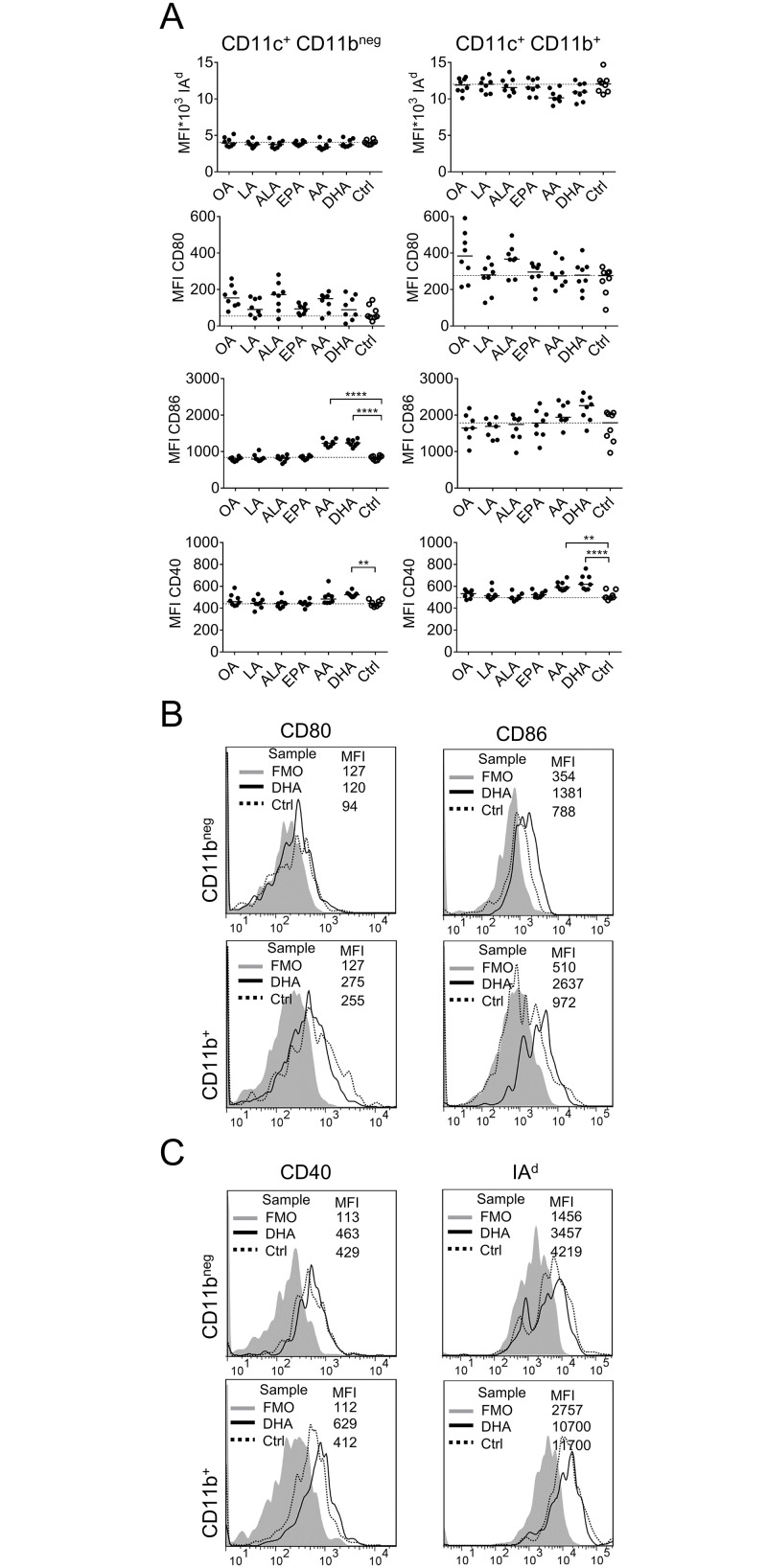
Expression of MHC class II and costimulatory molecules on fatty-acid supplemented dendritic cells (DCs). DC cultures were supplemented with fatty acids (50 μM); α-linolenic acid (ALA), arachidonic acid (AA), eicosapentaenoic acid (EPA), docosahexaenoic acid (DHA), linoleic acid (LA), oleic acid (OA) or ethanol only (Ctrl) for 3 days and thereafter analyzed by flow cytometry. (A) Mean fluorescence intensity (MFI) of IA^d^, CD80, CD86 and CD40. Left panel show MFI of CD11c^+^CD11b^neg^ and right panel on CD11c^+^CD11b^+^ DCs. Each dot represents one individual. Black dots denote samples supplemented with fatty acid while white dots with black borders denote control (ethanol only). Horizontal solid black lines show median value. The median from the control group has been extended with a dotted line for easy comparison to the other groups. Statistical mean difference was compared to the control group. Data are representative of two independent experiments. (B) Representative histograms for CD80 and CD86. (C) Representative histograms for CD40 and IA^d^. In the histograms FMO samples are shown with grey color, DHA samples are transparent with solid black border and control samples (ethanol only) are transparent with dotted black border. p-values: ** <0.01, *** <0.001, **** <0.0001.

**Fig 4:**
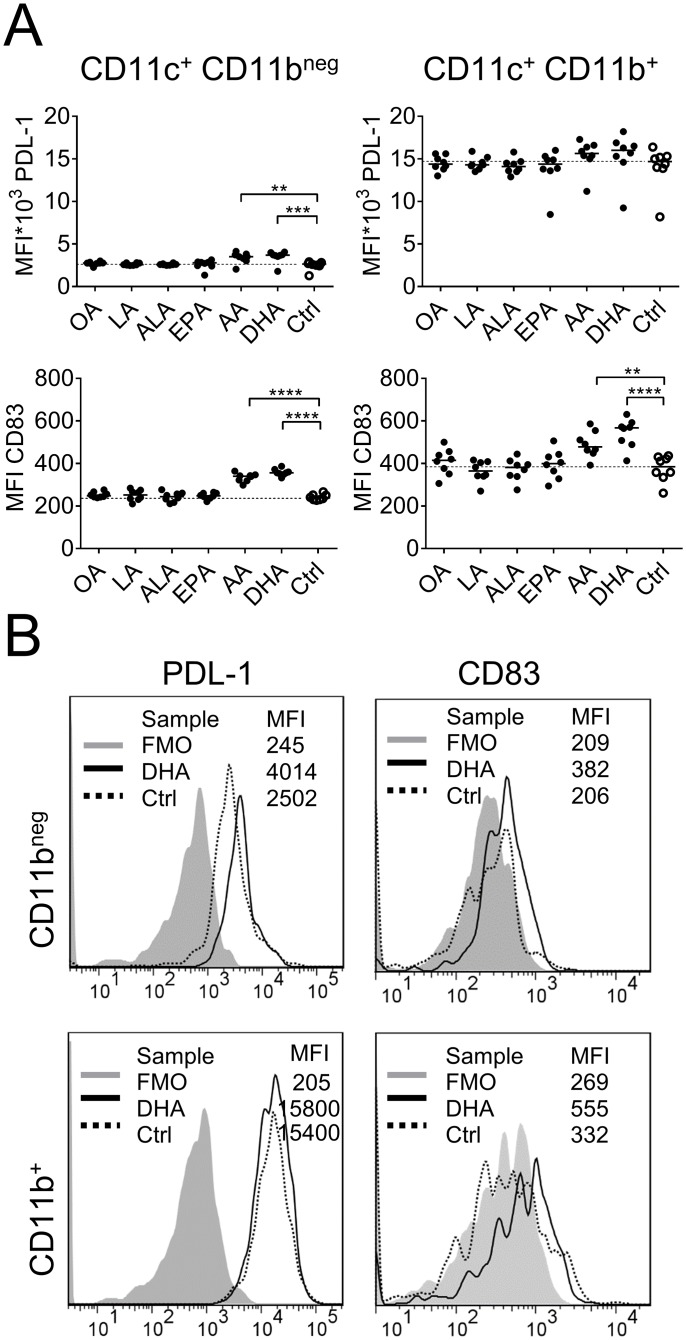
Expression of programmed death ligand-1 (PDL-1) and CD83 on fatty-acid supplemented dendritic cells (DCs). DC cultures were supplemented with fatty acids (50 μM); α-linolenic acid (ALA), arachidonic acid (AA), eicosapentaenoic acid (EPA), docosahexaenoic acid (DHA), linoleic acid (LA), oleic acid (OA) or ethanol only (Ctrl) for 3 days and thereafter analyzed by flow cytometry. (A) Mean fluorescence intensity (MFI) of PDL-1 and CD83. Left panel show MFI of CD11c^+^CD11b^neg^ and right panel of CD11c^+^CD11b^+^ DCs. Each dot represents one individual. Black dots denote samples supplemented with fatty acid while white dots with black borders denote control (ethanol only). Horizontal solid black lines show median value. The median from the control group has been extended with a dotted line for easy comparison to the other groups. Statistical mean difference was compared to the control group. Data are representative of two independent experiments. (B) Representative histogram of each marker. In the histograms FMO samples are shown with grey color, DHA samples are transparent with solid black border and control samples (ethanol only) are transparent with dotted black border. p-values: ** <0.01, *** <0.001, **** <0.0001.

After 3 days of *in vitro* culture with fatty acids DCs were analyzed by flow cytometry for separation of DC subsets and examination of their costimulatory molecule expression (Experimental design, S1 Fig). The FACS analysis revealed a high background in both the CD11c and CD11b channel (Fig 2A), reflecting low purity and pronounced autofluorescence of *in vitro* cultured cells. CD11c^+^ cells were gated, based on higher fluorescence than to the FMO control (Fig 2A) and divided into CD11c^+^ CD11b^+^ (upper gate) and CD11c^+^ CD11b^neg^ (lower gate) subpopulations, shown in Fig 2A. This was made because the relative level of expression for several cell surface molecules were higher in the CD11b^+^ group, as seen in the histograms in Figs 3 and 4. The proportion of CD11c^+^ cells (Fig 2B) or CD11c^+^ CD11b^+^ cells (Fig 2C) did not differ between cultures supplemented with fatty acids, compared to control cultures with ethanol only.

### Increase of costimulatory molecules on DCs after culture with arachidonic acid or DHA

We investigated whether supplementation with fatty acids affected DC expression of MHC class II (IA^d^) and costimulatory molecules, CD80, CD86 and CD40. As seen in Fig 3A, expression of MHC class II, i.e. IA^d^, was unaffected by fatty acids, only a non-significant reduction was observed with arachidonic acid. Supplementation with oleic acid or α-linolenic acid induced non-significantly higher CD80 expression (Fig 3A). In contrast, CD86 was significantly up-regulated on CD11c^+^ CD11b^neg^ DCs in response to arachidonic acid and DHA, and also tended to increase on CD11c^+^ CD11b^+^ cells in response to DHA (Fig 3A). CD40 expression was up-regulated on CD11c^+^ CD11b^+^ in cultures with arachidonic acid or DHA, and on CD11c^+^ CD11b^neg^ DCs in cultures with DHA (Fig 3A). Representative histograms of MFI values for CD80 and CD86 are shown in Fig 3B and for CD40 and IA^d^ in Fig 3C.

The effect of fatty acid supplementation on expression of the inhibitory molecule PDL-1 and the DC maturation marker CD83 is shown in Fig 4A. CD83 was significantly increased on DCs cultured with arachidonic acid or DHA, which correlated with up-regulation of CD86 (r = 0.9266, p< 0.0001 for CD11c^+^ CD11b^+^ DCs and r = 0.8465, p< 0.0001 for CD11c^+^ CD11b^neg^ DCs, S3 Fig). Also PDL-1 was up-regulated with arachidonic acid and DHA, although only significant for the CD11c^+^ CD11b^neg^ DCs (Fig 4A). Representative histograms of MFI values for CD83 and PDL-1 are shown in Fig 4B.

### Generation of prostaglandin E_2_ in arachidonic acid-primed dendritic cell cultures

**Fig 5:**
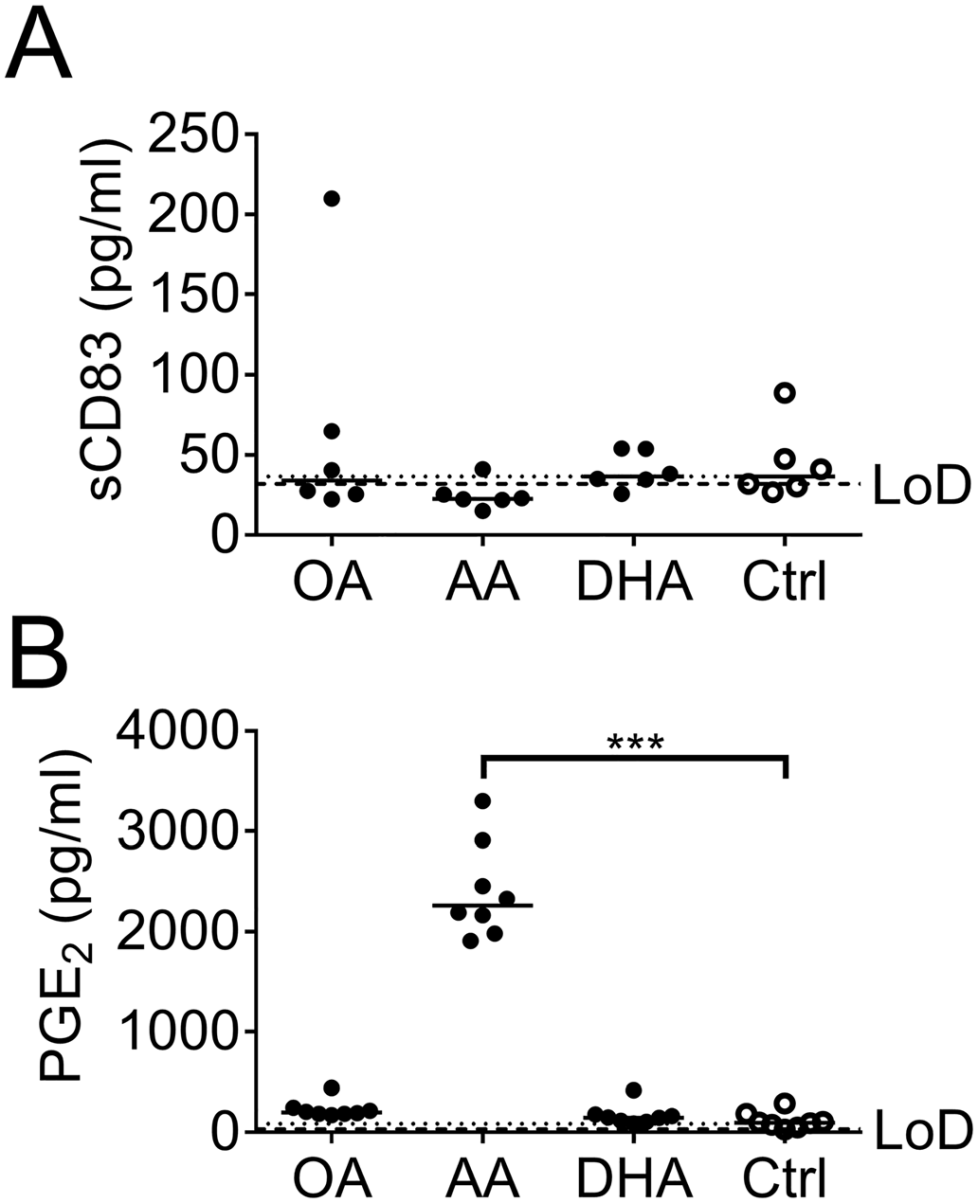
Soluble CD83 (sCD83) and prostaglandin E_2_ (PGE_2_) in dendritic cell (DC) culture supernatant. (A) Levels of sCD83 (pg/ml). (B) Levels of PGE_2_ (pg/ml). DC cultures were supplemented with fatty acids (50 μM); arachidonic acid (AA), docosahexaenoic acid (DHA), oleic acid (OA) or ethanol (Ctrl) for 3 days and supernatants analyzed by ELISA. Black dots denote samples supplemented with fatty acid while white dots with black borders denote control (ethanol only). Horizontal solid black lines show median value. The median from the control group has been extended with a dotted line for easy comparison to the other groups. Statistical mean difference was compared to the control group. Data are representative of one experiment. The limit of detection (LoD) is shown with dashed black lines. p-values: * <0.05, ** <0.01, *** <0.001.

We also measured soluble factors in the supernatant of DC cultures stimulated with the fatty acids arachidonic acid, DHA or oleic acid for 72 hours. We observed high levels of PGE_2_, >2 pg/ml, in DC cultures stimulated with arachidonic acid whereas <0.2 pg/ml of PGE_2_ was found with the other stimuli (Fig 5B). Since we observed an upregulation of CD83 expression on DCs supplemented with arachidonic acid and DHA we also measured soluble CD83 (sCD83) in the supernatant. Only low levels of sCD83 was detected, and without differences between the groups (Fig 5A). The levels of IL-10, IL-12 p70, and IFNγ were below detection limit while TGF-β1 levels were in pair with the levels in fresh complete IMDM (containing fetal bovine serum), with no differences between the groups.

In conclusion, MHC expression on DCs was unaffected by fatty acids, while arachidonic acid and DHA up-regulated the costimulatory molecules CD40, CD83, CD86 and the inhibitory molecule PDL-1. However, arachidonic acid distinguished itself from the other fatty acids in its ability to induce PGE_2_ production.

### Dendritic cells cultured with arachidonic acid or DHA reduced T-cell proliferation

**Fig 6:**
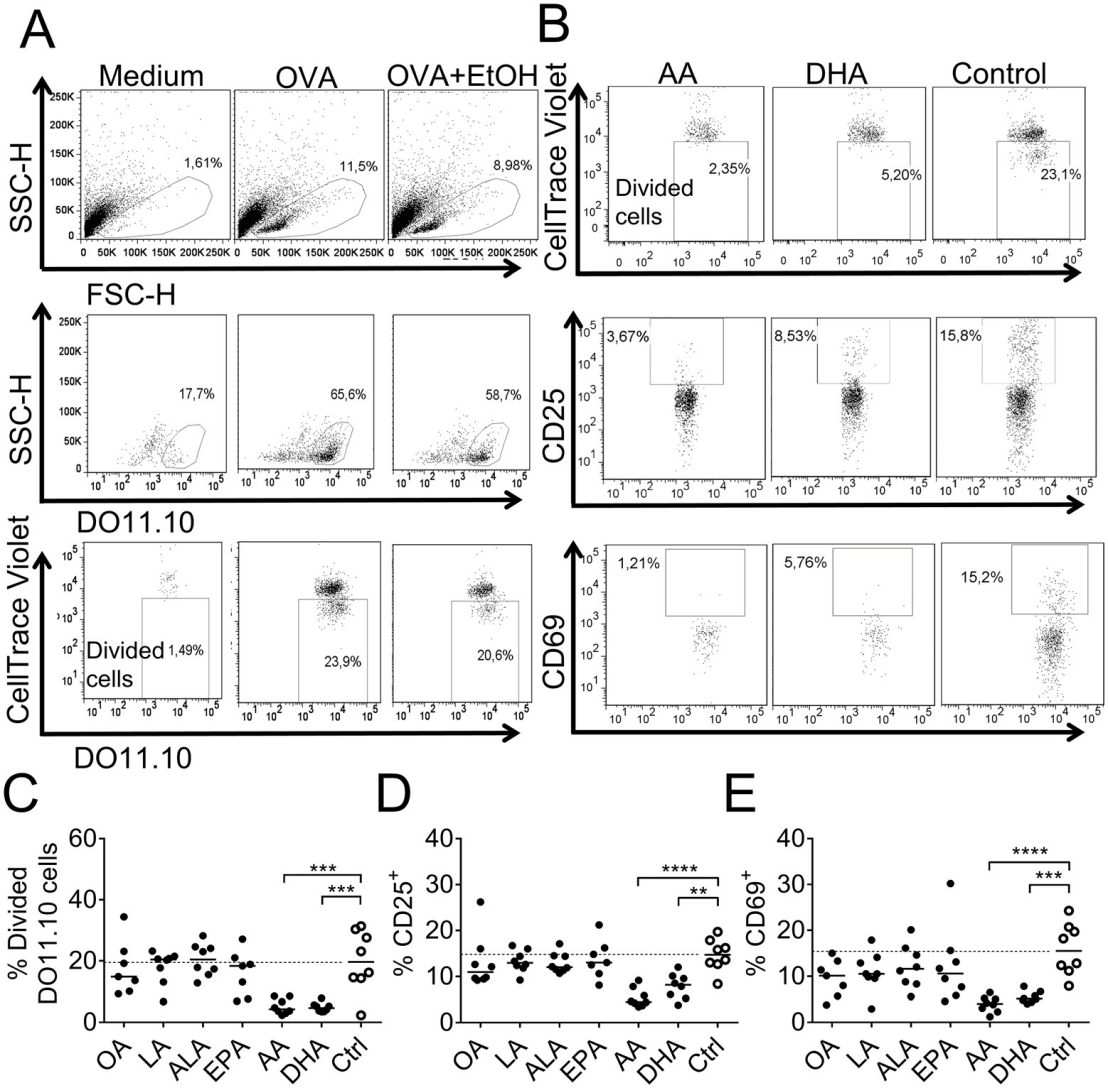
Activation of T cells by fatty-acid supplemented dendritic cells (DCs). T cells were co-cultured for 6 days with DCs previously supplemented with fatty acids (50 μM); α-linolenic acid (ALA), arachidonic acid (AA), eicosapentaenoic acid (EPA), docosahexaenoic acid (DHA), linoleic acid (LA), oleic acid (OA) or ethanol only (Ctrl); and thereafter analyzed by flow cytometry. (A) Gating strategy for lymphocytes (upper row), DO11.10^+^ T cells (middle row) and divided DO11.10^+^ T cells; CellTrace^™^ Violet^low^ (bottom row). (B) Representative dot plots showing dividing and activated (CD25^+^ and CD69^+^) DO11.10^+^ cells for arachidonic acid (AA), DHA and control samples. (C) Proportion divided, (D) CD25^+^ and (E) CD69^+^ of DO11.10^+^ T cells. Each dot represents one individual. Black dots denote samples supplemented with fatty acid while white dots with black borders denote control (ethanol only). Horizontal solid black lines show median value. The median from the control group has been extended with a dotted line for easy comparison to the other groups. Statistical mean difference was compared to the control group. p-values: ** <0.01, *** <0.001, **** <0.0001.

Next, we investigated if the fatty acid exposure of DCs would alter the outcome of the antigen-presentation to naïve T cells. Therefore, DCs were cultured with fatty acid and the model antigen OVA for 3 days, thereafter OVA-specific DO11.10^+^ T cells were added to the DC cultures (S1 Fig). The gating strategy of T cells is shown in Fig 6A and 6B. Briefly, lymphocytes were gated (upper 3 panels, Fig 6A), followed by gating for DO11.10 expression (middle panel, Fig 6A). Proliferation is shown in the lower panel of Fig 6A. After culture with OVA, about 25% of the T cells had divided, only 2% had divided in the absence of antigen. The solvent, ethanol had an inhibitory effect on proliferation, i.e. about 20% of the T cells proliferated in control cultures (Fig 6A). Arachidonic acid- or DHA-primed DCs reduced subsequent T-cell proliferation, compared to the control DCs, from ≈ 20% to ≈ 5% (Fig 6C). Expression of the activation markers CD69 and CD25 was reduced in parallel (Fig 6D and 6E). There was a correlation between proliferation and expression of CD69 (r = 0.8587, p < 0.0001) as well as proliferation and CD25 (r = 0.8935, p < 0.0001) (S4 Fig). None of the other investigated fatty acids (linoleic acid, α-linolenic acid, oleic acid or EPA) suppressed the capacity to activate T cells.

### Cell death in T-cell cultures with arachidonic acid- or DHA-primed dendritic cells

**Fig 7:**
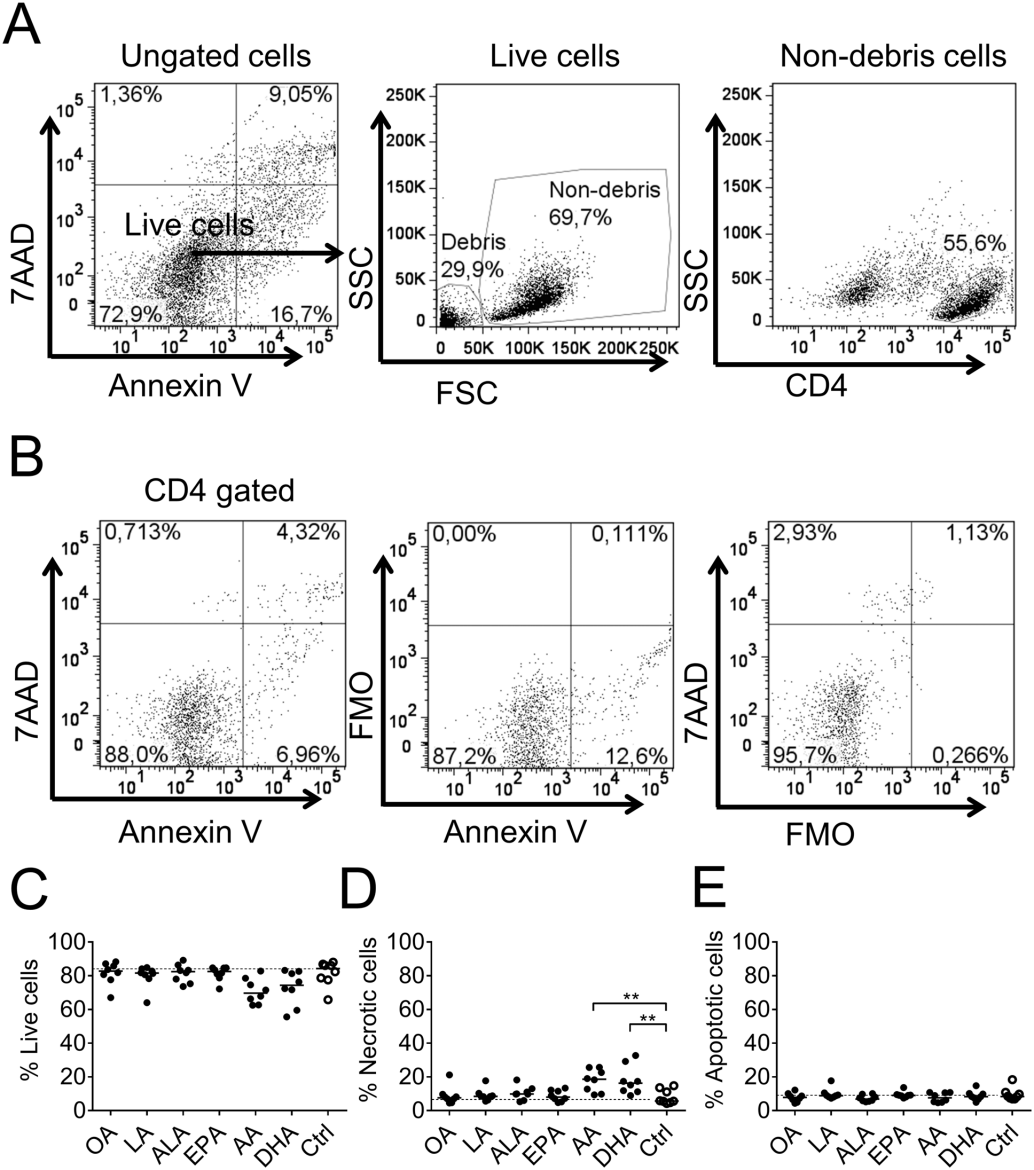
Viability of T cells after co-culture. T cells were analyzed with 7AAD and Annexin V after 6 days of co-culture with dendritic cells (DCs) previously supplemented with fatty acids (50 μM); α-linolenic acid (ALA), arachidonic acid (AA), eicosapentaenoic acid (EPA), docosahexaenoic acid (DHA), linoleic acid (LA), oleic acid (OA) or ethanol only (Ctrl). (A) Stepwise gating procedure to eliminate debris. (B) Representative dot plots showing gating of live cells (Annexin V^-^ 7AAD^-^), apoptotic cells (Annexin V^+^ 7AAD^-^) and necrotic and late apoptotic cells (Annexin V^+^ 7AAD^+^). (C) Proportion of live, (D) necrotic and late apoptotic and (E) apoptotic CD4^+^ T cells. Each dot represents one individual. Black dots denote samples supplemented with fatty acid while white dots with black borders denote control (ethanol only). Horizontal solid black lines show median value. The median from the control group has been extended with a dotted line for easy comparison to the other groups. Statistical mean difference was compared to the control group. Data are representative of two independent experiments. p-values: ** <0.01, *** <0.001, **** <0.0001.

We determined the levels of apoptosis and necrosis in the T cells by staining with Annexin V and 7AAD. The gating strategy is shown in Fig 7A and 7B. Briefly, to identify debris, live cells (Annexin V^-^ 7AAD^-^) were gated and on those cells we put a non-debris gate from which CD4^+^ T cells were gated for Annexin V and 7AAD staining. There was a non-significantly lower proportion of live T cells in the DC: T cell co-cultures with arachidonic acid-primed DCs compared to control cultures (70% vs 80%, Fig 7C) and a corresponding increase in late stage apoptotic/necrotic cells (Annexin V^+^ 7AAD^+^, ≈ 20% compared to 5%, Fig 7D), but no differences in early stage apoptotic T cells (Annexin V^+^ 7AAD^-^, Fig 7E).

### Regulatory T cells in cultures with arachidonic acid- or DHA-primed dendritic cells

**Fig 8:**
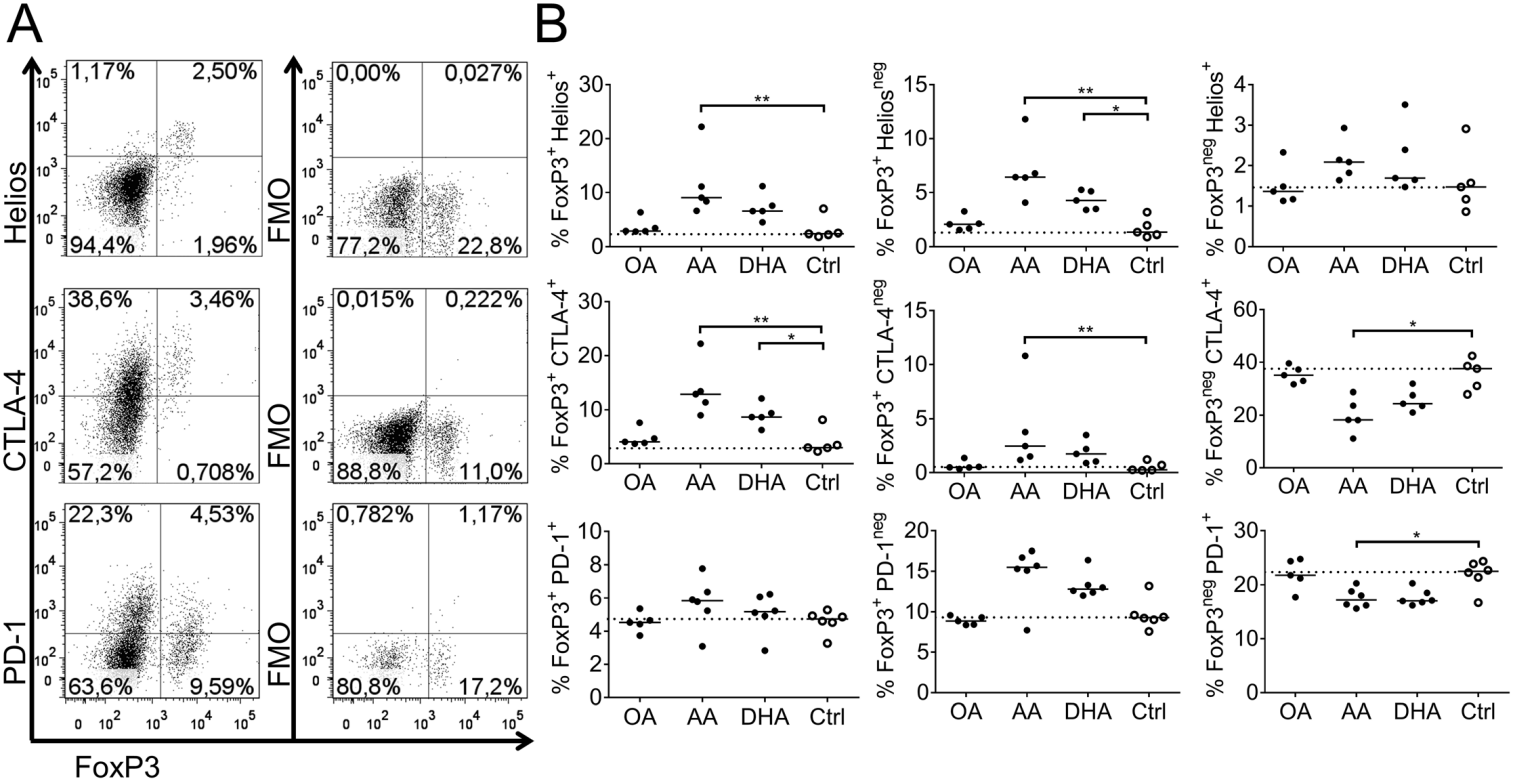
Co-expression of FoxP3 with Helios, CTLA-4 and PD-1. T cells were co-cultured for 6 days with dendritic cells (DCs) previously supplemented with fatty acids (50 μM); arachidonic acid (AA), docosahexaenoic acid (DHA), oleic acid (OA) or ethanol only (Ctrl); and thereafter analyzed by flow cytometry. (A) Representative dot plots from control samples showing staining of FoxP3^+^ and indicated markers (left column). In each plot double-positive-cells are found in the upper right quadrant and double-negative cells in the lower left quadrant. FMO samples are shown in the right column. (B) Proportion of double-positive (right column), FoxP3^+^ samples (middle column) and FoxP3^-^ samples (left column) in CD4^+^ cells. Each dot represents one individual. Black dots denote samples supplemented with fatty acid while white dots with black borders denote control (ethanol only). Horizontal black solid lines show median value. The median from the control group has been extended with a dotted line for easy comparison to the other groups. Statistical mean difference was compared to the control group. Data are representative of two independent experiments. p-values: * <0.05, ** <0.01, *** <0.001, **** <0.0001.

Since arachidonic acid and DHA were the only fatty acids that affected DC phenotype as well as the DCs’ ability to induce T-cell proliferation and activation markers, we chose to focus on these and included oleic acid as an inert fatty acid control. To investigate if the low T-cell proliferation observed with arachidonic acid- and DHA-primed DCs could be linked to presence of Tregs in the cultures we stained the T cells with FoxP3, Helios, CTLA-4 and PD-1. For representative dot plots and gating strategy see Fig 8A. We found that the proportion of FoxP3^+^ DO11.10^+^ T cells, either co-expressing Helios, CTLA-4 or PD-1 or not, were higher in DC: T cell co-cultures in which the DCs had been primed with arachidonic acid, and to some extent also DHA, compared to control cultures (Fig 8B). In contrast the proportion of FoxP3^neg^ DO11.10^+^ T cells co-expressing CTLA-4 or PD-1 was lower (Fig 8B). With oleic acid-primed DCs the proportion of putative Tregs was similar as in the control cultures. If proliferation mainly occurred among effector T cells, and not Tregs, the higher proportion of Tregs with arachidonic acid and DHA might be due to limited proliferation in these cultures and not because of regulatory activity. Expression of Helios, CTLA-4 and PD-1 on the whole T cell population is shown in S5 Fig.

**Fig 9:**
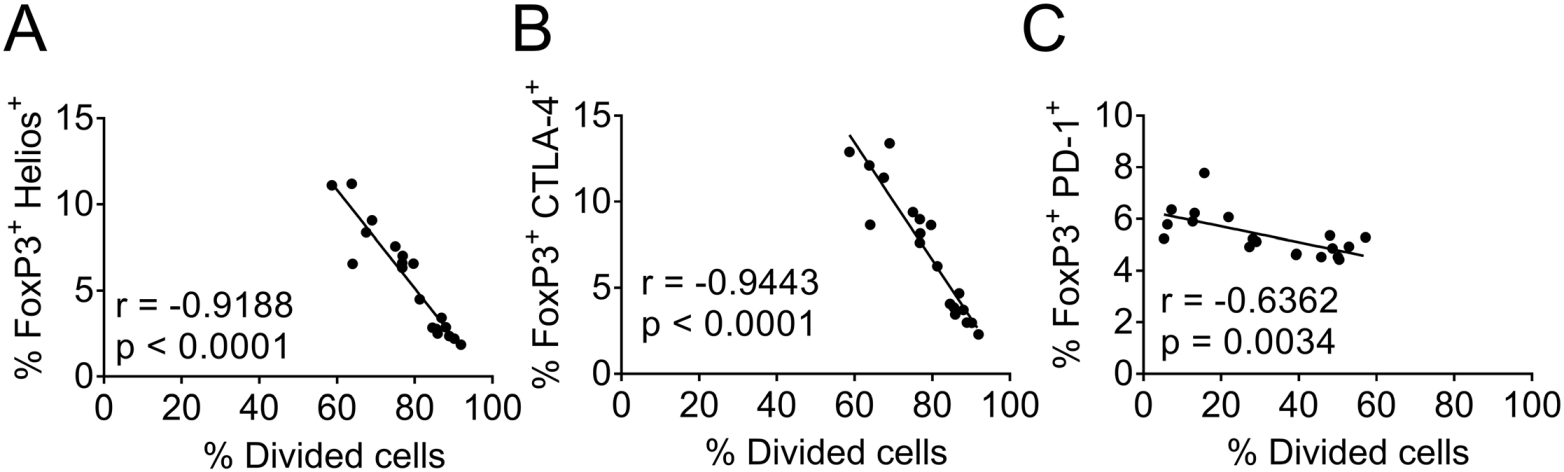
Correlation between proliferation and expression of regulatory markers on T cells. T cells were co-cultured for 6 days with dendritic cells (DCs) previously supplemented with fatty acids (50 μM); arachidonic acid (AA), docosahexaenoic acid (DHA), oleic acid (OA) or ethanol only (Ctrl); and thereafter analyzed by flow cytometry. Proportion of divided cells was correlated to proportion of T cells double-positive for FoxP3 and (A) Helios, (B) CTLA-4, and (C) PD-1. All samples, regardless of stimuli, were used in the same correlation analysis. The proportion of divided cells is different in (C) compared to (A) and (B) since this staining was performed in another experiment. r value: non-parametric Spearman correlation.

In addition we divided the DO11.10^+^ T cells into FoxP3^+^ and FoxP3^neg^ cells (S6A Fig) and as stated above, there was a significantly higher proportion of FoxP3^+^cells with both arachidonic acid and DHA relative to control (S6B Fig). The FoxP3^+^ T cells did not differ in MFI for FoxP3 depending on fatty acid-priming of the co-cultured DC (S6C Fig), meaning that arachidonic acid and DHA resulted in a higher proportion of FoxP3^+^ cells but not a higher expression of FoxP3 molecules on each cell. We further determined the expression of Helios, CTLA-4 and PD-1 by proportion (%) and MFI on both the FoxP3^+^ (S6D Fig) and FoxP3^neg^ T-cell population (S6E Fig). Phenotypically the FoxP3^+^ population were similar in all cultures i.e. the MFI values and proportions of Helios, CTLA-4 and PD-1 were similar between the different fatty acids and control (S6D and S6E Fig). As noted above, CTLA-4 was expressed to a lower extent in the FoxP3^neg^ population in both arachidonic and DHA compared to control cultures (S6E Fig). There was a negative correlation between proliferation (% divided cells) and proportion of Tregs assessed by the following populations; FoxP3^+^ Helios^+^ (Spearman r-value -0.92, p<0.0001), FoxP3^+^ CTLA-4^+^ (r = - 0.94, p<0.0001) and FoxP3^+^ PD-1^+^ (r = -0.63, p = 0.0034) (Fig 9). In summary, DCs supplemented with arachidonic acid and, to some extent, DHA inhibit T-cell proliferation, accompanied by a higher proportion of Tregs. These Tregs have a similar phenotype as Tregs found in oleic acid or control cultures.

### Lower levels of IL-10 and IFNγ with arachidonic acid-primed DCs in DC: T cell co-cultures

**Fig 10:**
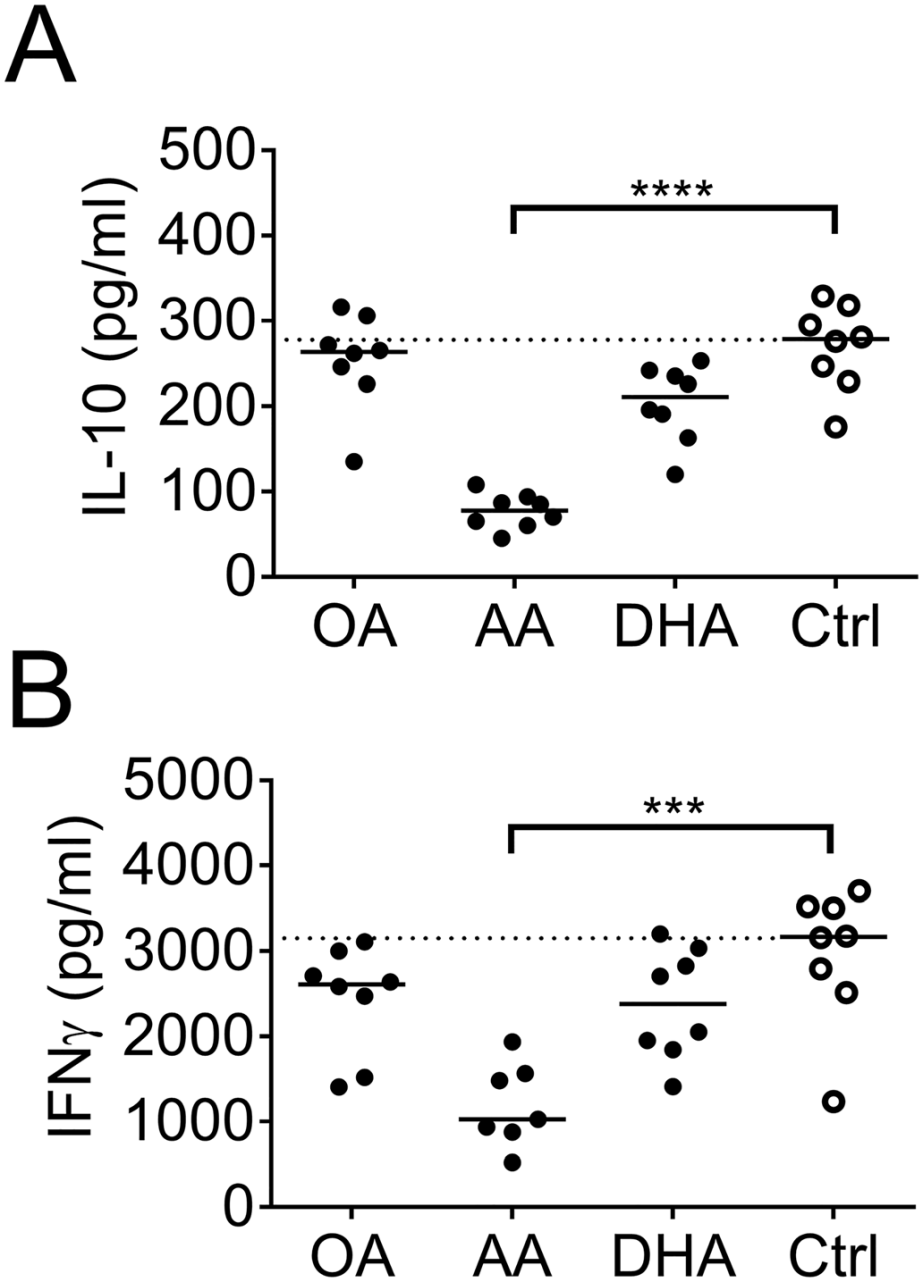
Cytokines in co-culture supernatant. Levels of (A) IL-10 and (B) IFNγ in DC: T cell co-culture supernatants. T cells were co-cultured for 6 days with dendritic cells (DCs) previously supplemented with fatty acids (50 μM); arachidonic acid (AA), docosahexaenoic acid (DHA), oleic acid (OA) or ethanol only (Ctrl). Each dot represents one individual. Horizontal black solid lines show median value. The median from the control group has been extended with a dotted line for easy comparison to the other groups. Statistical mean difference was compared to the control group. Data are representative of two independent experiments. p-values: * <0.05, ** <0.01, *** <0.001, **** <0.0001.

The DC: T cell co-culture supernatant from samples with arachidonic acid-primed DCs contained lower levels of IL-10 and IFNγ compared to control (Fig 10A and 10B), which supports the low T-cell stimulatory activity with arachidonic acid. No reduction of cytokines was shown with DHA-primed DCs, compared to controls, which suggests less T-cell suppression (Fig 10A and 10B). IL-12 p70 was below limit of detection, TGF-β1 was in pair with the levels in fresh complete IMDM, with no differences between the groups, while sCD83 was found in higher levels than those found in DC culture supernatant, but still without differences between the groups (S7 Fig).

### The PD-1: PDL1 pathway is partly responsible for the low proliferation in DC: T cell co-cultures with arachidonic acid- or DHA-primed DCs

**Fig 11:**
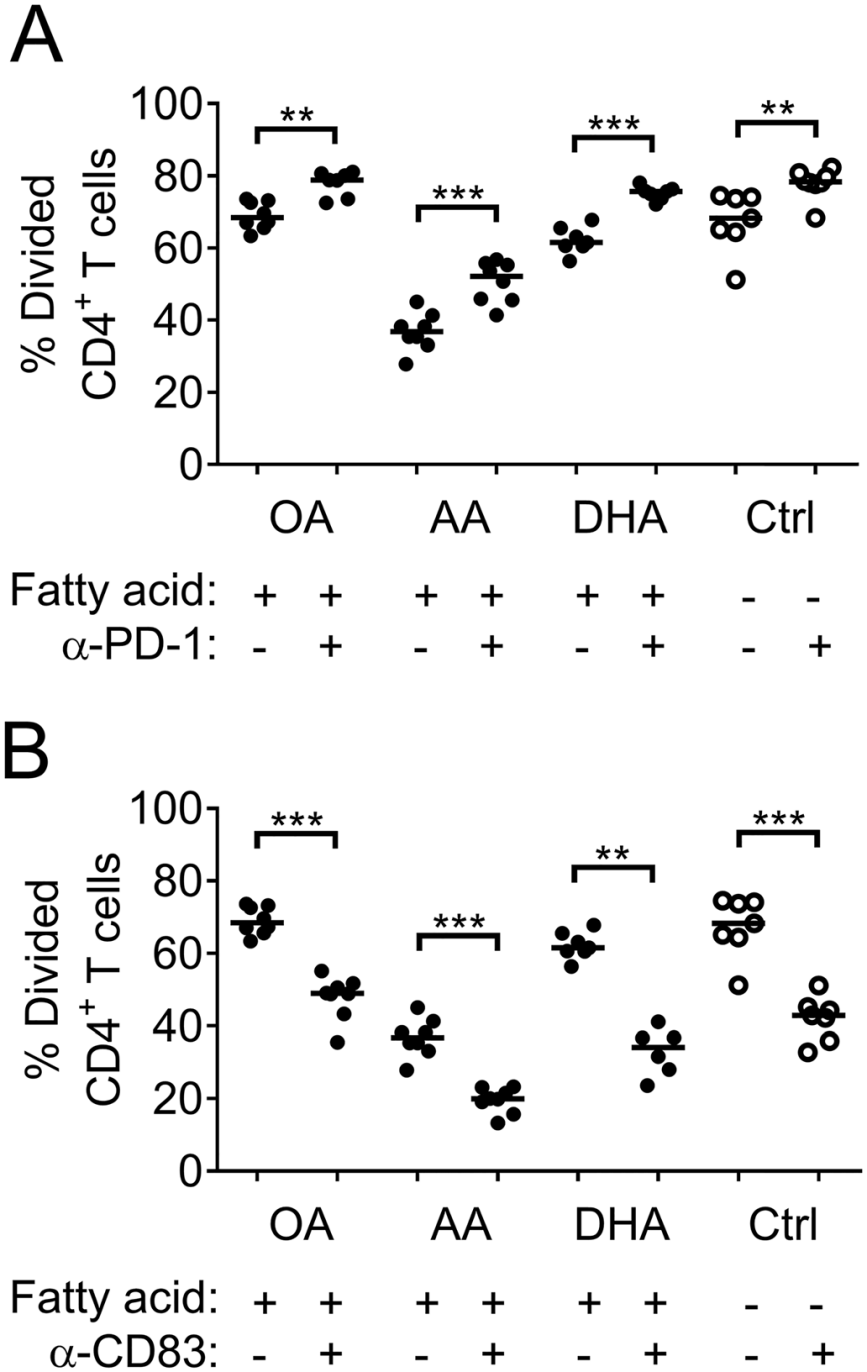
Proliferation of CD4^+^ T cells with and without blocking of CD83 or PD-1 during DC: T cell co-culture. T cells were co-cultured for 6 days with DCs previously supplemented with fatty acids (50 μM); arachidonic acid (AA), docosahexaenoic acid (DHA), oleic acid (OA) or ethanol only (Ctrl) without (-, No blocking) or with 10 μg/ml purified CD83-antibody (α-CD83) or PD-1 antibody (α-PD-1); and thereafter analyzed by flow cytometry. (A) Proportion of T cells that has proliferated (CellTrace^™^ CFSE^low^), with (+) or without (-) PD-1 blocking. (B) Proportion of T cells that has proliferated (CellTrace^™^ CFSE^low^), with (+) or without (-) CD83 blocking. Each dot represents one individual. Black dots denote samples supplemented with fatty acid while white dots with black borders denote control (ethanol only). Horizontal black solid lines show median value. Statistical mean difference, for each fatty acid or control, was compared between no blocking and blocking. Data are representative of two experiment. p-values: * <0.05, ** <0.01, *** <0.001, **** <0.0001.

DCs supplemented with arachidonic acid or DHA had increased expression of PDL-1 and CD83. To evaluate if these markers were involved in the reduced T-cell activation induced by such DCs we added either anti-CD83 or anti-PD-1 blocking antibodies to the DC: T-cell co-cultures. To achieve a more efficient blocking we blocked PD-1 instead of PDL-1, because PD-1 can also bind another molecule, PDL-2. As demonstrated in Fig 11 blocking of PD-1 led to increased proliferation in all DC: T-cell co-cultures. Blocking of PD-1 in arachidonic acid cultures did not restore proliferation to control levels, indicating that other pathways are involved in the low T-cell response. Blocking of CD83 led to lower proliferation in all DC: T-cell co-cultures. This supports the literature describing CD83 as an activation [35] and maturation marker [36].

The PD-1: PDL-1 pathway promotes induction of Tregs [37], so therefore we tested if PD-1 blocking changed the Treg phenotype or functionality. Indeed, the proportion of FoxP3^+^ Helios^+^ T cells decreased (S8A Fig) while the FoxP3^+^ Helios^neg^ population was unaffected (S8B Fig). The putative inhibitory population of FoxP3^+^ CTLA-4^+^ T cells was unaffected as well, with exception for the control group (S8C Fig). Hence, PD-1 blocking in fatty acid supplemented cultures decreased the proportion of FoxP3^+^ Helios^+^. In this experiment there was a negative correlation between FoxP3^+^ Helios^+^ T cells and proliferation (r = -0.7161, p < 0.0001) without blocking. With PD-1 blocking this negative correlation was increased (r = -0.7680, p < 0.0001). Fisher r-to-z transformation revealed that this change in r-value between PD-1 blocking and no blocking was statistically non-significant (z = 0.43, p (two-tailed) = 0.6672). Hence, with PD-1 blocking, the suppressive capacity of the FoxP3^+^ Helios^+^ T cells was unaffected, but the proliferation was increased, possibly due to a lower proportion of FoxP3^+^ Helios^+^ T cells.

## Discussion

In the present study we tested the effect of six fatty acids on expression of MHC class II and costimulatory molecules on DCs and on the ability of the DCs to induce proliferation of naïve T cells in the presence of antigen. Culture of DCs with the long-chain PUFAs arachidonic acid or DHA, but no other fatty acid, led to increased expression of CD40, CD83 and CD86 as well as PDL-1 on the DC. Strikingly, however, DCs exposed to arachidonic acid or DHA induced lower antigen-specific T-cell proliferation and activation compared to DCs cultured in control medium. In parallel, a lower proportion of naïve T cells co-cultured with arachidonic acid- or DHA-primed DCs expressed the activation markers CD25 and CD69. Thus, despite a mature phenotype, DCs supplemented with the n-6 PUFA arachidonic acid or the n-3 PUFA DHA, did not activate T cells.

Tregs, that express the lineage-specific transcription factor FOXP3 are known to prevent effector T-cell proliferation [38]. Immunosuppressive effects of fatty acids mediated by induction of Tregs have been obtained by EPA supplementation during transplantation [39] and by dietary intake of arachidonic acid and DHA, together but not one by one, in a mouse model of dermatitis [40]. DC expression of the costimulatory molecules CD40, CD80 and CD86, has been inversely associated with generation of FoxP3^+^ T cells from naïve CD4^+^ T cells [41] but in our study the number of FoxP3^+^ Helios^+^ DO11.10^+^ T cells were higher after supplementation with arachidonic acid or DHA to DCs, despite up-regulated activation markers. CTLA-4 is an inhibitory molecule important for Tregs, stored intracellular, but mobilized to the surface upon activation [42] where it binds CD80 or CD86. It has been reported as necessary for generation of Tregs *in vitro* [43]. In our study CTLA-4, measured intracellular, was not up-regulated in the T-cell cultures with suppressed proliferation. However our DC: T-cell co-culture lasted for 6 days, and Linsley *et al*. [44] have reported that surface expression of CTLA-4 peaks 48 h after activation and returns to normal levels after additionally 48 hours. Altogether, if the decreased T-cell proliferation was due to induction of Tregs, and the higher proportion of Tregs was not the result of limited T-cell proliferation, we have shown that arachidonic acid and DHA can induce FoxP3^+^ Helios^+^ Tregs *in vitro* via DCs.

PD-1: PDL-1 interaction can inhibit T-cell proliferation and activation [23, 45] and could therefore be expected to be responsible for the T-cell unresponsiveness. Proposed mechanisms are induction of Tregs [37]. With arachidonic acid and DHA DCs had higher PDL-1 MFI than control DCs. However, the corresponding receptor PD-1 was not up-regulated on T cells. Our blocking experiment showed that PD-1 blocking decreased the proportion of FoxP3^+^ Helios^+^ T cells, which negatively correlated with increased proliferation. This effect was not limited to any of the stimuli. The FoxP3^+^ Helios^+^ subset is generally regarded as thymus derived so we question if these cells are generated *in vitro* or if they’re decreased due to higher proliferation of effector T cells. If so, FoxP3^+^ Helios^neg^ T cells has proliferated to the same extent as FoxP3^neg^ T cells, since PD-1 blocking did not change the proportion of these. Expression of PD-1 has also been associated with programmed cell death [46]. Therefore we analyzed T-cell viability with Annexin V and 7AAD. The level of apoptotic and necrotic cells was generally low, with reduced viability in cultures with arachidonic acid or DHA. This might be due to lack of proliferation, an induction of tolerance via cell death.

CD83 was up-regulated with arachidonic acid and DHA. To our knowledge no association between fatty acids and CD83 has been reported. The most established role for CD83 is involvement in maturation of CD4^+^ T cells in the thymus [47]. In different setups CD83 has been shown to support expansion of newly primed naïve CD8^+^ T cells [48], be required for lymphocyte longevity [35], enhance DCs’ stimulatory capacity [49] and thereby induce allogeneic T-cell proliferation [50]. However, the lack of a known ligand have hampered mechanistic explanations of CD83. CD83 expression has been correlated to expression of MHC class II [47, 51, 52] and CD86 [47, 52]. Here, up-regulation of CD83 correlated with up-regulation of CD86, and CD83 blocking resulted in decreased proliferation, suggesting a stimulatory role for CD83. CD83 is also found in soluble form, sCD83, formed via proteolytic cleavage of membrane-bound CD83 [53]. The extracellular soluble CD83 domain can inhibit DC-mediated T-cell proliferation [54] as well as inhibit T-cell proliferation and production of IL-2 and IFNγ *in vitro* [55, 56]. We measured sCD83 but found no differences between the groups, so this could not explain the T-cell unresponsiveness observed in cultures stimulated with arachidonic acid and DHA.

Rather than having an effect on cell membrane fluidity the effects of arachidonic acid and DHA can be caused by fatty-acid specific mechanisms. As free fatty acids, arachidonic acid and DHA are metabolically active compounds with ability to regulate intracellular signaling pathways and gene transcription but also serve as precursors for lipid mediators involved in inflammation. However, the suppressive effect of PUFAs can be independent of lipid mediators, as blocking of conversion enzymes in DCs not necessarily limit suppression of T-cell activation [25]. Because of the involvement in intracellular signaling the levels of free fatty acids are regulated on several levels. Most fatty acids are esterified and bound in the membrane or in triglycerides. Carrier proteins, such as fatty acid binding proteins (FABPs), can increase the solubility and regulate the accessibility of the fatty acids to their target molecules. PUFAs can affect expression for a plethora of genes, including the nuclear receptor peroxisome proliferator-activating receptors (PPARs) [57]. PPARγ is up-regulated in DCs upon differentiation [58] and its expression can be induced by DHA [32]. PPARγ induction reduces DC expression of CD40, CD80 and CD86 and capacity to activate naïve CD4^+^ T cells *in vitro*, as shown by decreased IL-2 and IFNγ production [59].

Other studies have determined the lipid content of DCs and the association to antigen presenting capacity. However the results are contradictory, DCs with high lipid content, in comparison to DCs with low lipid content, have been shown to be both tolerogenic [60] but also pro-immunogenic [61]. In a study by Herber *et al*. MHC molecules, CD40, CD80, CD86 and DC-SIGN were unaffected by triglyceride content while in a study by Ibrahim *et al*. lipid-laden DCs had higher expression of CD1d, CD40, CD54, CD80 and CD86. Our DCs, stimulated with arachidonic acid or DHA, displayed an activated phenotype but yet seemed to act in a more tolerogenic fashion resulting in low T-cell responses. We did not measure total lipid content of our DCs but it might be that total fatty acid content is not the parameter that drives DCs to become more or less tolerogenic. Instead ratios between different fatty acids or content of individual fatty acids might be the immunomodulatory parameter. Arachidonic acid had a stronger suppressive capacity than DHA and lowered the levels of IL-10 and IFNγ, but gave higher DC culture supernatant levels of PGE_2_. Our result is in line with the notion that long-chain PUFAs reduce T-cell proliferation, but we can show that this is true not only when the fatty acids are added to the T cells but also when supplemented to DCs in an earlier cell culture. The induction of FoxP3^+^ Tregs warrants further studies of the capability of these Tregs to suppress other T cells.

## Supporting Information

S1 Fig Experimental design. Dendritic cells (DCs) were isolated based on CD11c expression and cultured *in vitro* with fatty acids dissolved in ethanol with or without OVA. DCs in medium without OVA were characterized with flow cytometry at day 3 while those with OVA were washed and thereafter co-cultured with OVA-specific T cells, stained with CellTrace^™^ Violet, for 6 additional days prior to flow cytometry analysis of T cells at day 9.
(TIF)

S2 Fig Purity of fatty acids. Stock solutions of arachidonic acid (AA), docosahexaenoic acid (DHA), oleic acid (OA) and ethanol (99% solution) were analyzed with gas chromatography, with and without internal control. Purity is reported as area under curve of indicated fatty acid divided with area under curve for all identified peaks. Note the difference in abundance on the y axis for ethanol compared to the other samples.
(TIF)

S3 Fig The correlation between expression of CD83 and CD86 on fatty-acid supplemented dendritic cells (DCs). DC cultures were supplemented with fatty acids (50 μM); α-linolenic acid (ALA), arachidonic acid (AA), eicosapentaenoic acid (EPA), docosahexaenoic acid (DHA), linoleic acid (LA), oleic acid (OA) or ethanol only (Ctrl) for 3 days and thereafter analyzed by flow cytometry. Proportion of DCs expressing CD83 was correlated to expression of CD86 for (A) CD11c^+^CD11b^+^ DCs and (B) CD11c^+^CD11b^neg^ DCs. All samples, regardless of stimuli, were used in the same correlation analysis. Data was tested for normality and correlation computed with Pearson correlation test.
(TIF)

S4 Fig The correlation between proliferation and expression of CD69 and CD25. T cells for 6 days were co-cultured with dendritic cells (DCs) previously supplemented with fatty acids; arachidonic acid (AA), docosahexaenoic acid (DHA), oleic acid (OA) or ethanol (Ctrl); and thereafter analyzed by flow cytometry. Proportion of divided cells (proliferation) was correlated to expression of (A) CD69 and (B) CD25. Data was tested for normality (D'Agostino & Pearson omnibus normality test) and correlation computed with Pearson correlation test (CD25) or Spearman correlation test (CD69).
(TIF)

S5 Fig Expression of Helios, CTLA-4 and PD-1. T cells were co-cultured for 6 days with dendritic cells (DCs) previously supplemented with fatty acids (50 μM); arachidonic acid (AA), docosahexaenoic acid (DHA), oleic acid (OA) or ethanol only (Ctrl); and thereafter analyzed by flow cytometry. Expression of (A) Helios, (B) CTLA-4 and (C) PD-1. Each dot represents one individual. Black dots denote samples supplemented with fatty acid while white dots with black borders denote control (ethanol only). Horizontal black solid lines show median value. The median from the control group has been extended with a dotted line for easy comparison to the other groups. Statistical mean difference was compared to the control group. Data are representative of two independent experiments. p-values: * <0.05, ** <0.01, *** <0.001, **** <0.0001.
(TIF)

S6 Fig Phenotype of FoxP3^+^ and FoxP3^neg^ T cells. T cells were co-cultured for 6 days with dendritic cells (DCs) previously supplemented with fatty acids (50 μM); arachidonic acid (AA), docosahexaenoic acid (DHA), oleic acid (OA) or ethanol only (Ctrl); and thereafter analyzed by flow cytometry. (A) Gating strategy for FoxP3^+^ (upper gate) and FoxP3^neg^ (lower gate) DO11.10^+^ T cells. (B) Proportion of FoxP3^+^ DO11.10^+^ T cells. (C) Mean fluorescence intensity (MFI) of FoxP3 for the FoxP3^+^ T cells shown in (B). Phenotype of FoxP3^+^ (D) and FoxP3^neg^ (E) DO11.10^+^ T cells. For both groups proportion of CTLA-4^+^, Helios^+^ and PD-1^+^ cells are shown in the left column and MFI of the same markers in the right column. Each dot represents one individual. Black dots denote samples supplemented with fatty acid while white dots with black borders denote control (ethanol only). Horizontal solid black lines show median value. The median from the control group has been extended with a dotted line for easy comparison to the other groups. Statistical mean difference was compared to the control group. Data are representative of two independent experiments. p-values: * <0.05, ** <0.01, *** <0.001, **** <0.0001.
(TIF)

S7 Fig Soluble CD83 (sCD83) in DC: T cell co-culture supernatants. T cells were co-cultured with DCs previously supplemented with fatty acids; arachidonic acid (AA), docosahexaenoic acid (DHA), oleic acid (OA) or ethanol (Ctrl); after 3 days supernatants where taken and analyzed by ELISA. Each dot represents one individual. Horizontal black solid lines show median value. The median from the control group has been extended with a dotted line for easy comparison to the other groups. Statistical mean difference was compared to the control group. Data are representative of one experiment. The limit of detection (LoD) is shown with a dashed black line.
(TIF)

S8 Fig T regulatory subsets with and without blocking of PD-1 during DC: T cell co-culture. T cells were co-cultured for 6 days with DCs previously supplemented with fatty acids (50 μM); arachidonic acid (AA), docosahexaenoic acid (DHA), oleic acid (OA) or ethanol only (Ctrl) without (-) or with (+) 10 μg/ml purified PD-1 antibody (α-PD-1); and thereafter analyzed by flow cytometry. (A) Proportion of FoxP3^+^ Helios^+^ T cells. (B) Proportion of FoxP3^+^ Helios^neg^ T cells. (C) Proportion of FoxP3^+^ CTLA-4^+^ T cells. Each dot represents one individual. Black dots denote samples supplemented with fatty acid while white dots with black borders denote control (ethanol only). Horizontal black solid lines show median value. Statistical mean difference, for each fatty acid or control, was compared between no blocking and blocking. Data are representative of one experiment. p-values: * <0.05, ** <0.01, *** <0.001, **** <0.0001.
(TIF)
