# Supplementary figures and images for: The Polyunsaturated Fatty Acids Arachidonic Acid and Docosahexaenoic Acid Induce Mouse Dendritic Cells Maturation but Reduce T-Cell Responses In Vitro
Source: PLoS One. 2015 Nov 30;10(11):e0143741. doi: 10.1371/journal.pone.0143741 (PMC4664484; doi:10.1371/journal.pone.0143741)

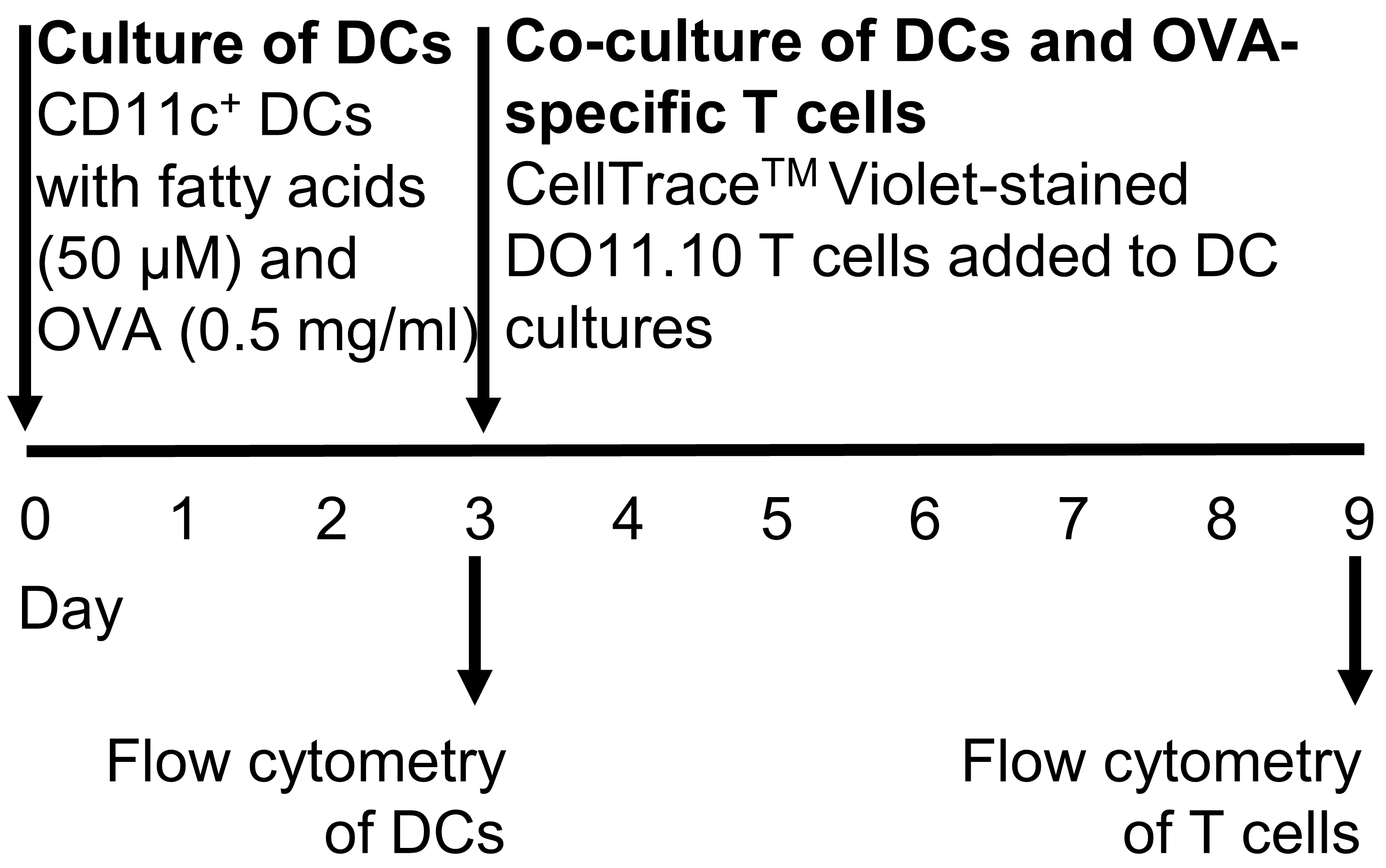

Supplement: S1 Fig — Dendritic cells (DCs) were isolated based on CD11c expression and cultured in vitro with fatty acids dissolved in ethanol with or without OVA. DCs in medium without OVA were characterized with flow cytometry at day 3 while those with OVA were washed and thereafter co-cultured with OVA-specific T cells, stained with CellTrace™ Violet, for 6 additional days prior to flow cytometry analysis of T cells at day 9. (TIF) [file pone.0143741.s001.tif]

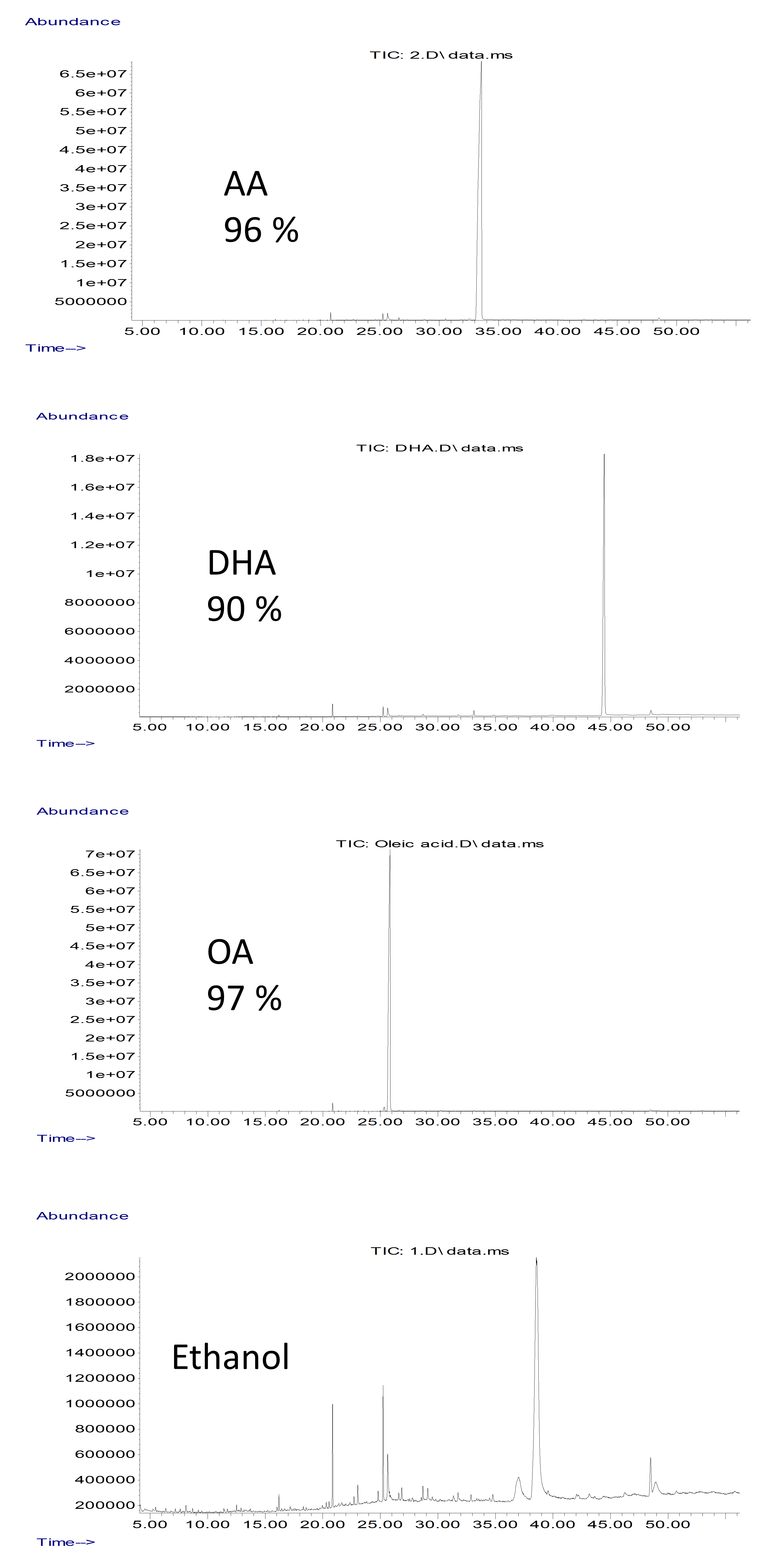

Supplement: S2 Fig — Stock solutions of arachidonic acid (AA), docosahexaenoic acid (DHA), oleic acid (OA) and ethanol (99% solution) were analyzed with gas chromatography, with and without internal control. Purity is reported as area under curve of indicated fatty acid divided with area under curve for all identified peaks. Note the difference in abundance on the y axis for ethanol compared to the other samples. (TIF) [file pone.0143741.s002.tif]

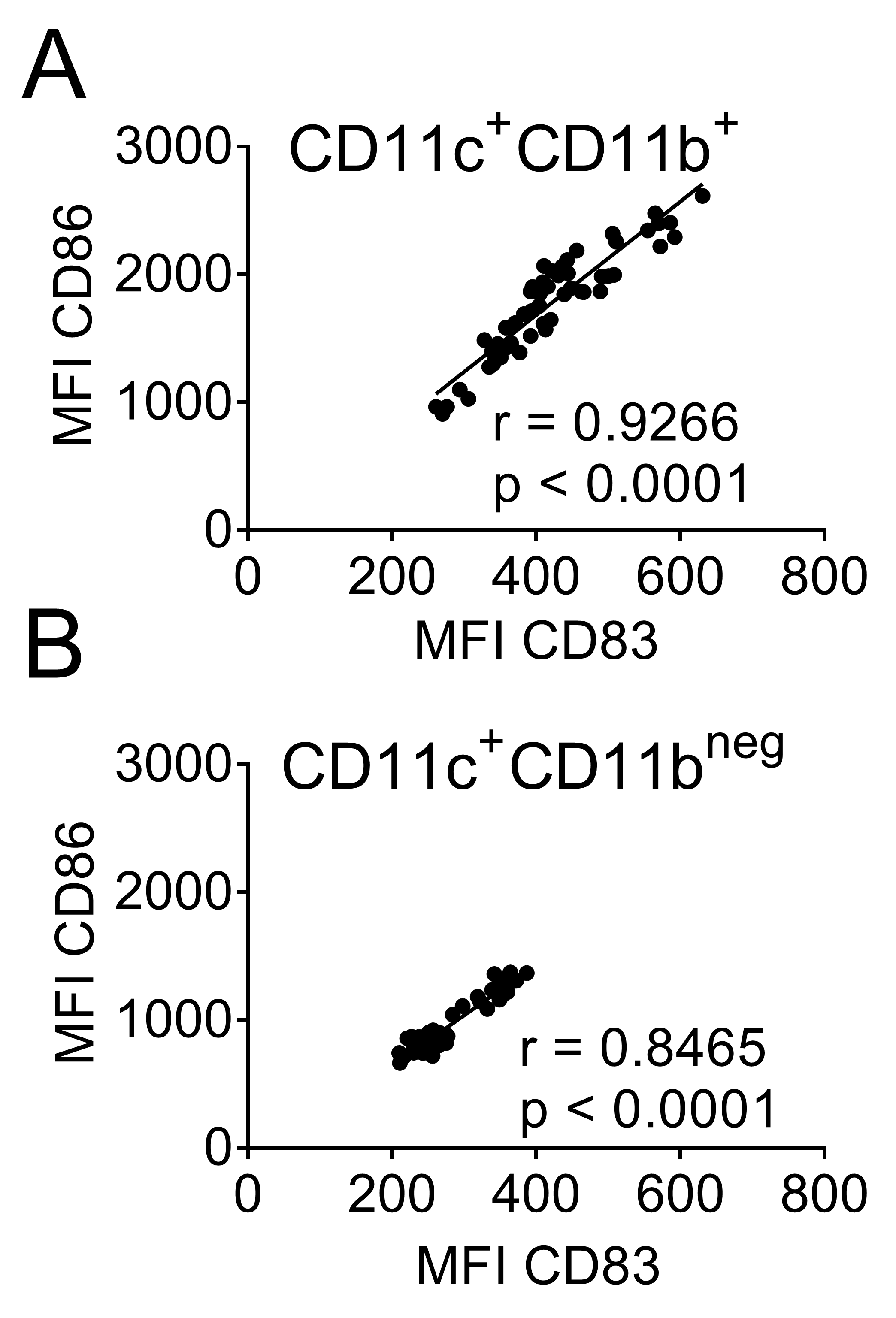

Supplement: S3 Fig — DC cultures were supplemented with fatty acids (50 μM); α-linolenic acid (ALA), arachidonic acid (AA), eicosapentaenoic acid (EPA), docosahexaenoic acid (DHA), linoleic acid (LA), oleic acid (OA) or ethanol only (Ctrl) for 3 days and thereafter analyzed by flow cytometry. Proportion of DCs expressing CD83 was correlated to expression of CD86 for (A) CD11c+CD11b+ DCs and (B) CD11c+CD11bneg DCs. All samples, regardless of stimuli, were used in the same correlation analysis. Data was tested for normality and correlation computed with Pearson correlation test. (TIF) [file pone.0143741.s003.tif]

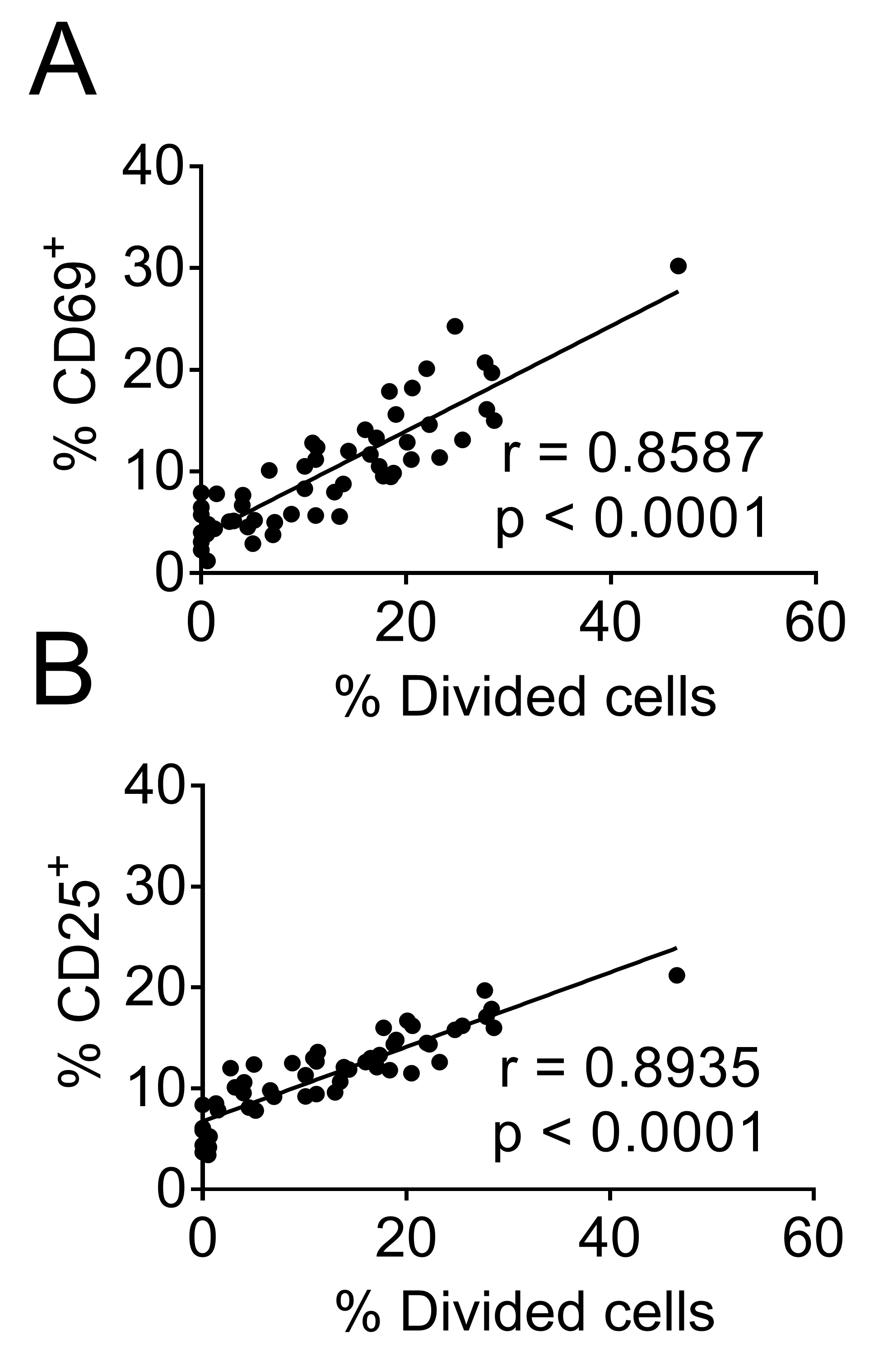

Supplement: S4 Fig — T cells for 6 days were co-cultured with dendritic cells (DCs) previously supplemented with fatty acids; arachidonic acid (AA), docosahexaenoic acid (DHA), oleic acid (OA) or ethanol (Ctrl); and thereafter analyzed by flow cytometry. Proportion of divided cells (proliferation) was correlated to expression of (A) CD69 and (B) CD25. Data was tested for normality (D'Agostino & Pearson omnibus normality test) and correlation computed with Pearson correlation test (CD25) or Spearman correlation test (CD69). (TIF) [file pone.0143741.s004.tif]

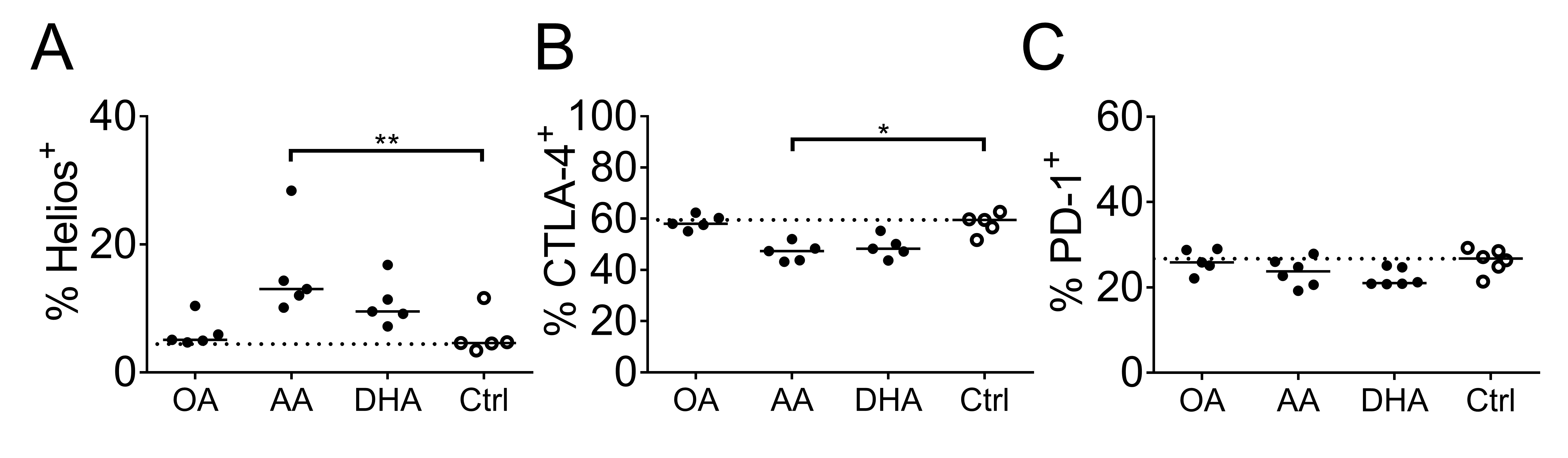

Supplement: S5 Fig — T cells were co-cultured for 6 days with dendritic cells (DCs) previously supplemented with fatty acids (50 μM); arachidonic acid (AA), docosahexaenoic acid (DHA), oleic acid (OA) or ethanol only (Ctrl); and thereafter analyzed by flow cytometry. Expression of (A) Helios, (B) CTLA-4 and (C) PD-1. Each dot represents one individual. Black dots denote samples supplemented with fatty acid while white dots with black borders denote control (ethanol only). Horizontal black solid lines show median value. The median from the control group has been extended with a dotted line for easy comparison to the other groups. Statistical mean difference was compared to the control group. Data are representative of two independent experiments. p-values: * <0.05, ** <0.01, *** <0.001, **** <0.0001. (TIF) [file pone.0143741.s005.tif]

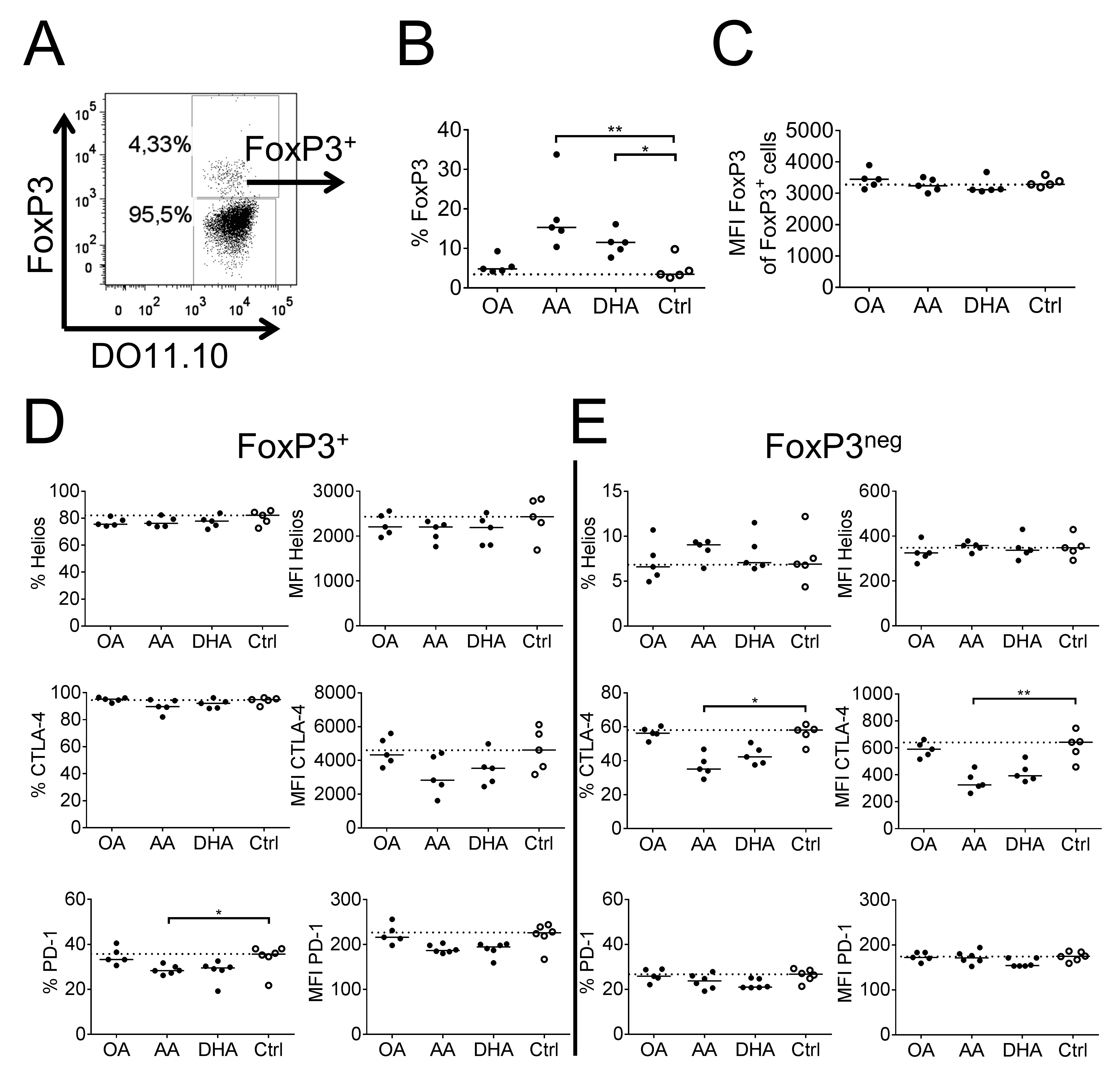

Supplement: S6 Fig — T cells were co-cultured for 6 days with dendritic cells (DCs) previously supplemented with fatty acids (50 μM); arachidonic acid (AA), docosahexaenoic acid (DHA), oleic acid (OA) or ethanol only (Ctrl); and thereafter analyzed by flow cytometry. (A) Gating strategy for FoxP3+ (upper gate) and FoxP3neg (lower gate) DO11.10+ T cells. (B) Proportion of FoxP3+ DO11.10+ T cells. (C) Mean fluorescence intensity (MFI) of FoxP3 for the FoxP3+ T cells shown in (B). Phenotype of FoxP3+ (D) and FoxP3neg (E) DO11.10+ T cells. For both groups proportion of CTLA-4+, Helios+ and PD-1+ cells are shown in the left column and MFI of the same markers in the right column. Each dot represents one individual. Black dots denote samples supplemented with fatty acid while white dots with black borders denote control (ethanol only). Horizontal solid black lines show median value. The median from the control group has been extended with a dotted line for easy comparison to the other groups. Statistical mean difference was compared to the control group. Data are representative of two independent experiments. p-values: * <0.05, ** <0.01, *** <0.001, **** <0.0001. (TIF) [file pone.0143741.s006.tif]

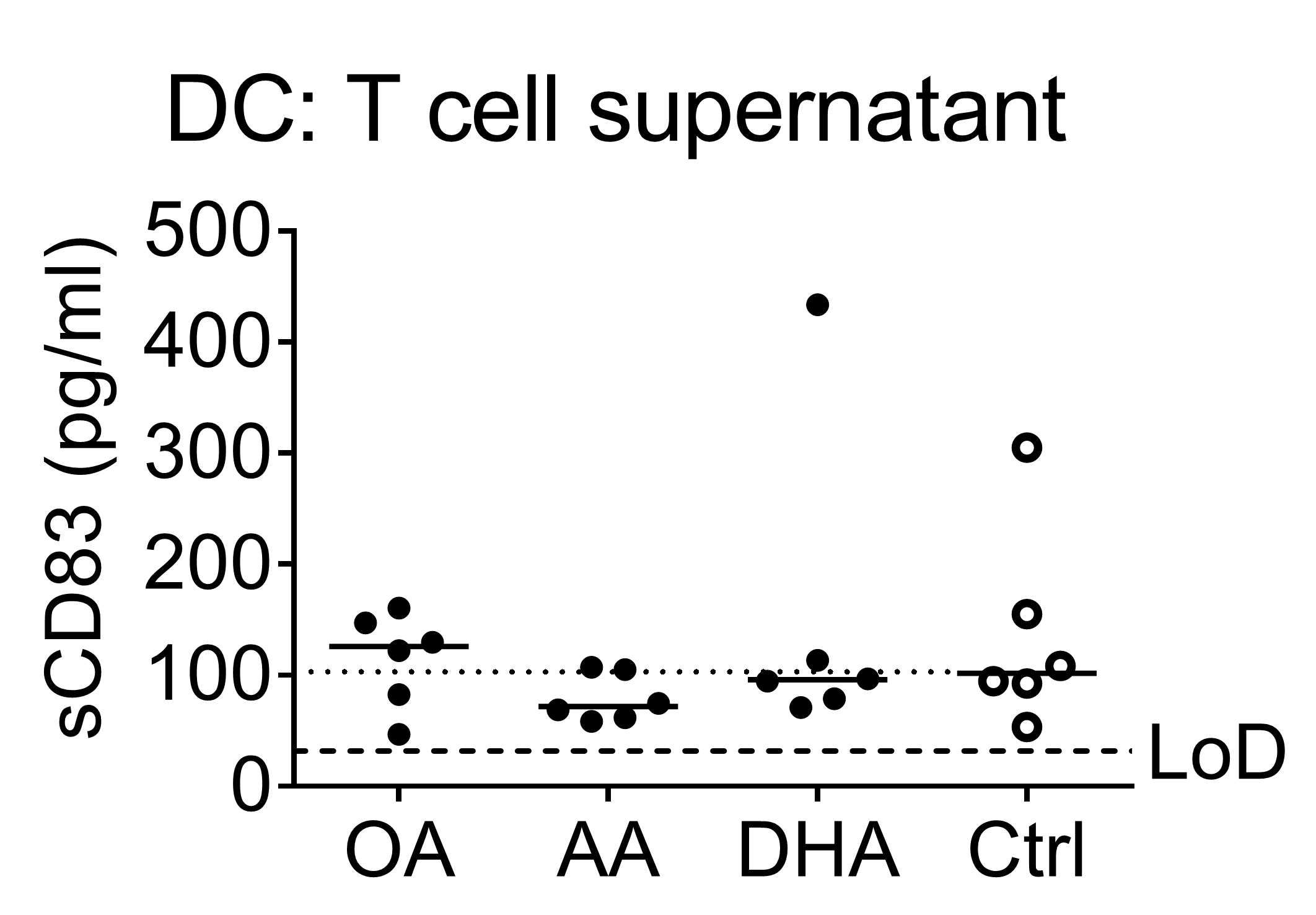

Supplement: S7 Fig — T cells were co-cultured with DCs previously supplemented with fatty acids; arachidonic acid (AA), docosahexaenoic acid (DHA), oleic acid (OA) or ethanol (Ctrl); after 3 days supernatants where taken and analyzed by ELISA. Each dot represents one individual. Horizontal black solid lines show median value. The median from the control group has been extended with a dotted line for easy comparison to the other groups. Statistical mean difference was compared to the control group. Data are representative of one experiment. The limit of detection (LoD) is shown with a dashed black line. (TIF) [file pone.0143741.s007.tif]

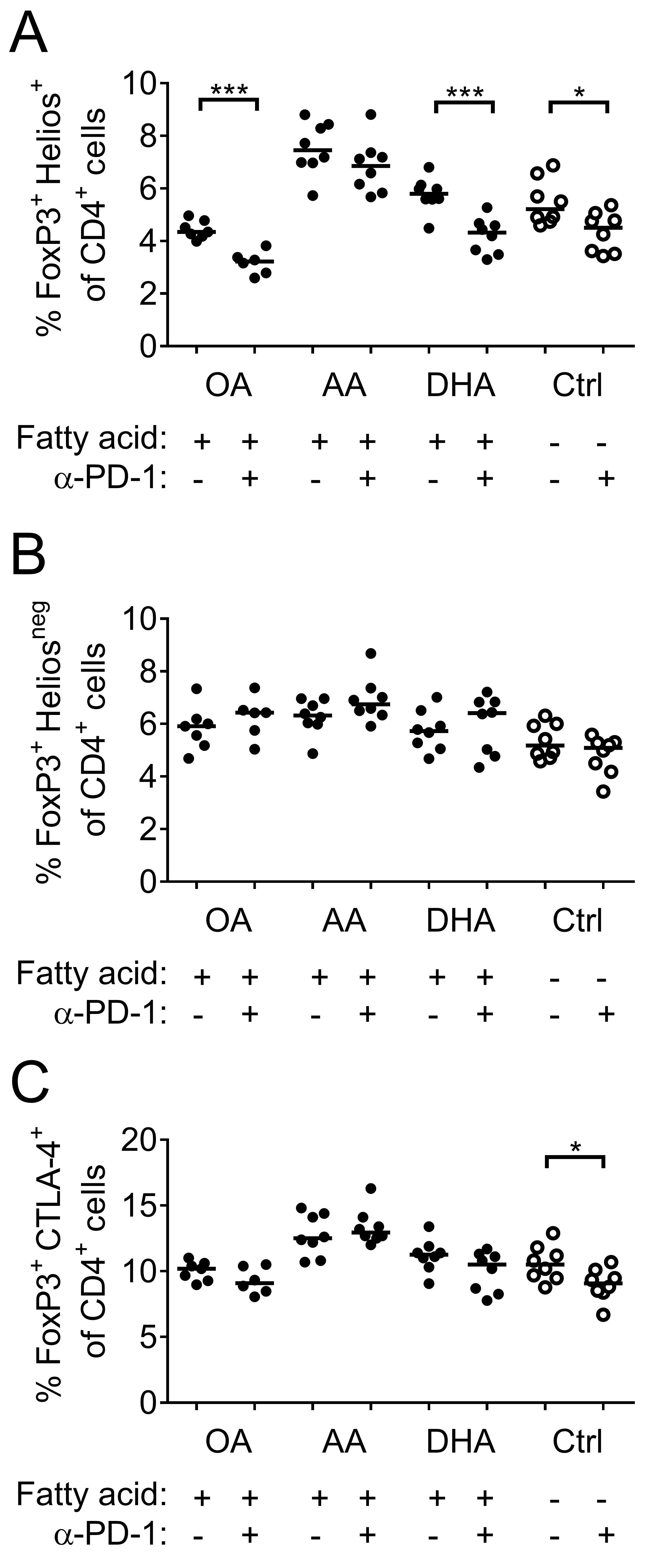

Supplement: S8 Fig — T cells were co-cultured for 6 days with DCs previously supplemented with fatty acids (50 μM); arachidonic acid (AA), docosahexaenoic acid (DHA), oleic acid (OA) or ethanol only (Ctrl) without (-) or with (+) 10 μg/ml purified PD-1 antibody (α-PD-1); and thereafter analyzed by flow cytometry. (A) Proportion of FoxP3+ Helios+ T cells. (B) Proportion of FoxP3+ Heliosneg T cells. (C) Proportion of FoxP3+ CTLA-4+ T cells. Each dot represents one individual. Black dots denote samples supplemented with fatty acid while white dots with black borders denote control (ethanol only). Horizontal black solid lines show median value. Statistical mean difference, for each fatty acid or control, was compared between no blocking and blocking. Data are representative of one experiment. p-values: * <0.05, ** <0.01, *** <0.001, **** <0.0001. (TIF) [file pone.0143741.s008.tif]
